# The diagnostic value of radiomics-based machine learning for lymph node metastasis in prostate cancer: a systematic review and meta-analysis

**DOI:** 10.3389/fonc.2026.1710716

**Published:** 2026-02-12

**Authors:** ZengHui Liu, Yin Yang, Xiaodong Guan

**Affiliations:** 1 First Clinical College, Changzhi Medical College, Changzhi, Shanxi, China; 2 Urinary Surgery, Yuncheng Central Hospital Affiliated to Shanxi Medical University, Yuncheng, Shanxi, China; 3 Clinical Discipline Development Center, Shanxi Medical University, Taiyuan, Shanxi, China; 4 Research Department, Yuncheng Central Hospital Affiliated to Shanxi Medical University, Yuncheng, Shanxi, China

**Keywords:** deep learning (DL), lymph node metastasis (LNM), machine learning (ML), prostate cancer (PC), radiomics

## Abstract

**Background:**

The precise and noninvasive diagnosis of preoperative lymph node metastasis (LNM) in prostate cancer (PC) is challenging. Some studies have studied the application of radiomics-based machine learning (ML) for detecting LNM in PC. However, systematic evidence regarding its diagnostic performance is still lacking.

**Aim:**

Our study aimed to systematically evaluate the accuracy of radiomics-based ML models in diagnosing LNM in PC, offering evidence-based support for the use of ML in clinical decision-making.

**Methods:**

Cochrane, PubMed, EMBASE, and Web of Science were searched for eligible studies on the diagnostic performance of radiomics-based ML for LNM in PC until June 11, 2025. The risk of bias in the included studies was evaluated via the Radiomics Quality Score (RQS). Meta-analysis of sensitivity (SEN) and specificity (SPC) was performed using a bivariate mixed-effects model. Subgroup analyses were performed in the meta-analysis based on imaging modality and modeling approach. We conducted meta-analysis on the training and validation sets, respectively.

**Results:**

A total of 22 studies were included, comprising 13 studies on positron emission tomography (PET)/computed tomography (CT)-based radiomics and nine studies on magnetic resonance imaging (MRI)-based radiomics. In the validation sets, models based on PET/CT yielded a pooled SEN of 0.89 (95% confidence interval (CI): 0.75–0.96), SPC of 0.82 (95% CI: 0.63–0.93), and a summary receiver operating characteristic (SROC) of 0.93 (95% CI: 0.77–0.98). Models based on MRI had a SEN of 0.84 (95% CI: 0.78–0.89), SPC of 0.86 (95% CI: 0.71–0.94), and a SROC of 0.90 (95% CI: 0.71–0.97). Radiomics-based ML models yielded a SEN of 0.85 (95% CI: 0.76–0.91), a SPC of 0.77 (95% CI: 0.66–0.86), and an area under the receiver operating characteristic (AUROC) of 0.89 (95% CI: 0.72–0.96). In contrast, deep learning (DL) models based on radiomics demonstrated a higher SEN of 0.88 (95% CI: 0.75–0.95), SPC of 0.97 (95% CI: 0.58–1.00), and a SROC of 0.95 (95% CI: 0.19–1.00).

**Conclusions:**

Radiomics demonstrates promising diagnostic performance in detecting LNM in PC. DL models show superior accuracy. Nevertheless, given the limited sample sizes, insufficient external validation, and heterogeneity in imaging protocols, future research should incorporate more multi-center images from different regions. Meanwhile, it is necessary to develop standardized imaging and segmentation protocols to improve transparency and reduce heterogeneity, thereby building more widely applicable and high-performance radiomics-based machine learning models to improve the performance of early detection of LNM in PC patients.

**Systematic Review Registration:**

https://www.crd.york.ac.uk/prospero/, identifier PROSPERO CRD420251085724.

## 1 Introduction

Prostate cancer (PC) represents the second most frequent malignancy in men worldwide (1, 2). As per GLOBOCAN 2022 of the International Agency for Research on Cancer (IARC), PC accounted for 397,000 deaths in 2022, representing 7.3% of male cancer-related mortality, and 1.47 million new cases, representing 14.7% of newly diagnosed malignancies in men. Its incidence is higher in developed countries (e.g., America and Europe), whereas its mortality burden is more pronounced in developing regions (e.g., Africa) (3). Consequently, PC has emerged as a major global health concern.

Lymph nodes constitute the second most frequent site of metastasis in PC (4). The traditional gold standard for confirming nodal metastasis is pelvic lymph node dissection (PLND), which provides histopathological evidence through surgical excision. However, this invasive procedure may fail to detect micrometastases and is only applicable to surgery candidates. Therefore, accurate lymph node staging is crucial for assessing patient prognosis, the risk of recurrence, and the potential for salvage therapy (5). Preoperative assessment of nodal status also has significant clinical implications for tailoring treatment strategies and avoiding unnecessary surgery or radiotherapy.

Currently, computed tomography (CT) and magnetic resonance imaging (MRI) are the primary imaging techniques for identifying lymph node metastasis (LNM) in PC. Nonetheless, their sensitivity (SEN) and specificity (SPC) are limited (6). The diagnostic performance of positron emission tomography (PET)/CT is approximately 27% higher than conventional imaging techniques (7). Nevertheless, the interpretation of imaging findings remains subject to inter-observer variability, which possibly introduces diagnostic bias. As machine learning (ML) rapidly develops, radiomics has demonstrated promising potential in the diagnosis and prognostic assessment of PC, including biochemical recurrence (8) and bone metastasis (9). ML, as a branch of artificial intelligence, can enable computer systems to learn automatically from data and discover patterns and then use these patterns to make predictions or decisions about new data, without relying on explicit, fixed instructions. To further enhance the precision and SEN of imaging-based assessments, a variety of predictive models based on radiomics have been developed to complement existing diagnostic modalities.

Radiomics, first introduced in 2012, is an emerging technique that enables us to extract high-throughput quantitative features from CT, MRI, and PET, among other medical imaging modalities. Radiomics can be combined with ML-based analyses to support disease diagnosis, prognostication, and therapeutic decision-making. A systematic review by Wen J et al. (10) has demonstrated that MR-based radiomics has favorable predictive accuracy in detecting extracystic prostatic expansion (EPE). A systematic review by Lomer NB et al. (11) has reported that MRI-based radiomics exhibited good performance in predicting the grade of PC. A study by Li Y et al. (12) shows that PSMA PET/CT demonstrates good performance in predicting the pathological progression of PC. Additionally, some studies have explored the use of radiomics-based ML in diagnosing LNM in PC.

However, differences in imaging modalities and modeling strategies have resulted in heterogeneity across radiomics-based predictive models for nodal status, and robust systematic evidence on their diagnostic performance is lacking. Therefore, our systematic review aimed to assess the diagnostic performance of radiomics-based models in the prediction of LNM in PC, thereby providing references for the development, research, and refinement of such models.

## 2 Methods

### 2.1 Study registration

This study followed Preferred Reporting Items for Systematic Reviews and Meta-Analyses of Diagnostic Test Accuracy (PRISMA DTA) and was registered in PROSPERO before commencement (registration no.: CRD420251085724).

### 2.2 Eligibility criteria

The inclusion criteria are as follows:

1. Studies involving patients with histologically confirmed PC.
2. Studies on radiomics-based models for assessing LNM in PC, including both traditional ML and deep learning (DL) algorithms. The images were segmented for learning and validation.
3. English publications.

The exclusion criteria include the following:

1. Unpublished conference abstracts.
2. Studies that only performed image segmentation without developing a radiomics-based model.
3. Studies with no outcome measures for assessing the diagnostic performance of ML models, like ROC, C-index, SEN, accuracy, recall, precision, SPC, contingency tables, F1 score, or calibration curves.

### 2.3 Data sources and search strategy

Cochrane, EMBASE, PubMed, and Web of Science were searched until June 11, 2025. Both subject andfree-text terms were utilized, without limitations on publication year or location. The search strategy is provided in **Supplementary Table S1**.

### 2.4 Study selection

All searched records were uploaded to EndNote for duplicate removal. Then, the titles and abstracts were read to exclude irrelevant studies. Subsequently, the full texts of possibly eligible articles were assessed. Two investigators (ZHL and YY) independently conducted the literature screening and then cross-checked their results. Dissents were addressed by a third investigator (XDG).

### 2.5 Data extraction

A standard form was created for data extraction. The extracted information encompassed title, first author, publication year, country, design, patient and radiomics sources, segmentation method, completeness of imaging protocol reporting, number of investigators involved in image segmentation, whether pilot studies under different imaging parameters were performed, whether test–retest studies were conducted, segmentation software, LNM cases in the entire cohort, total cases, LNM cases in the training and validation sets, training cases, validation set generation approach, presence of external validation, total validation cases, variable choosing approaches, model, modeling variables, construction of radiomics scores, code and data availability, as well as model performance metrics. Two investigators (ZHL and YY) independently extracted the data and then cross-checked their results. Dissents were addressed by a third investigator (XDG).

### 2.6 Study quality assessment

Study quality was assessed using the Radiomics Quality Score (RQS), a tool designed for assessing the quality of radiomics research. It encompasses 16 items within six domains. Two investigators (ZHL and YY) independently assessed the quality of the included studies and then cross-checked their results. Dissents were addressed by a third investigator (XDG).

### 2.7 Synthesis methods

Meta-analyses of SEN and SPC were carried out utilizing a bivariate mixed-effects model. When original studies did not report diagnostic 2 × 2 contingency tables, SEN, SPC, precision, and case numbers were used to derive the necessary data. The model was used to pool SEN, SPC, positive likelihood ratio (PLR), negative likelihood ratio (NLR), diagnostic odds ratio (DOR), and the summary receiver operating characteristic curve (SROC). If multiple models were constructed in an original study, we analyzed the model with the best accuracy in the validation set. Publication bias was detected using Deeks’ funnel plots. Subgroup analyses by imaging sources and model types (traditional ML versus DL) were carried out. All meta-analyses were conducted using Stata 15.0.

## 3 Results

### 3.1 Study selection

**Figure 1:**
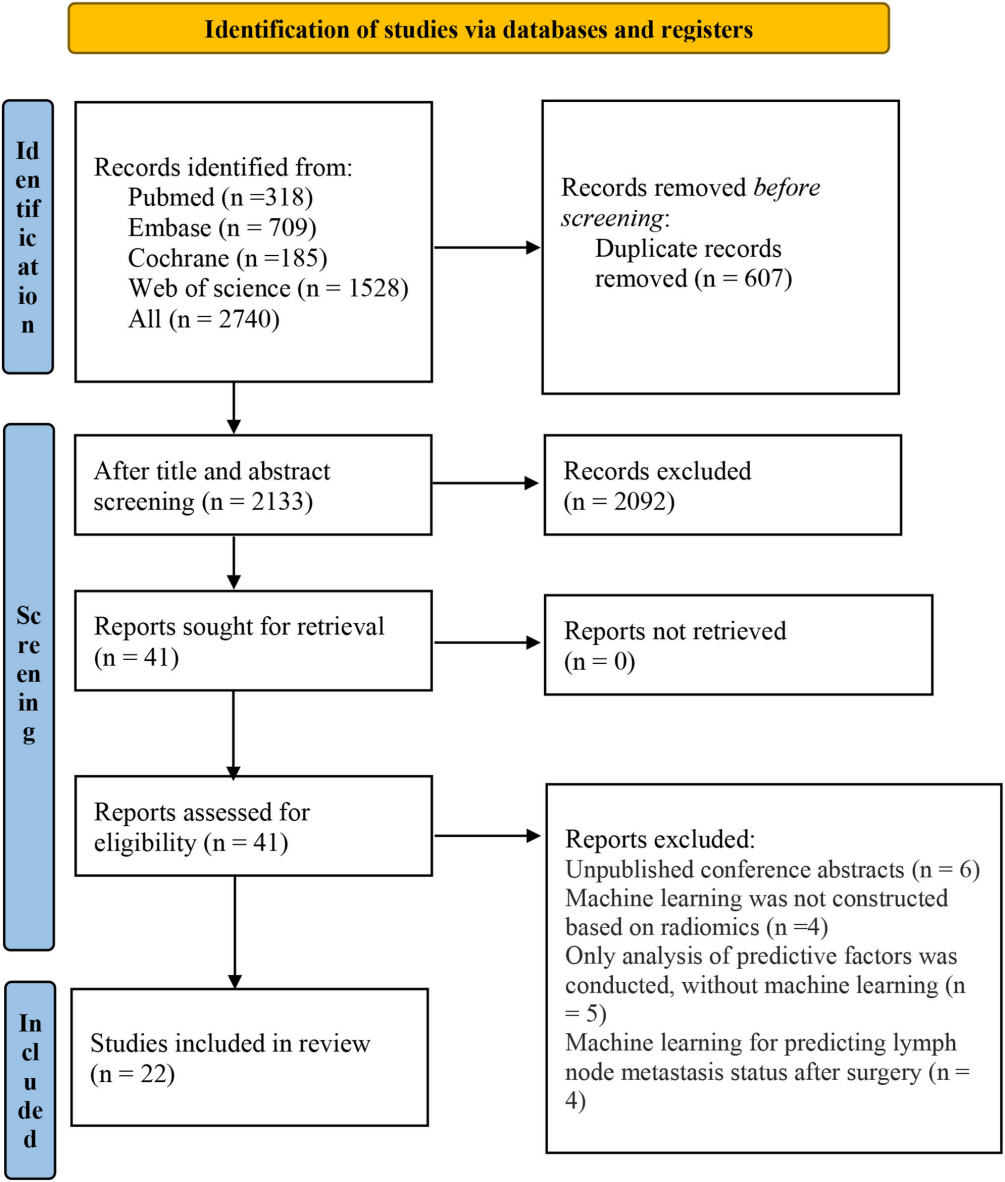
PRISMA flow diagram of study selection.

A total of 2,740 records were initially identified across the four databases. After removing duplicates, 2,133 studies remained for further screening. After reading titles and abstracts, 2,092 irrelevant studies were excluded. The full texts of the remaining 41 articles were reviewed. A total of 19 studies were excluded: six were conference abstracts without full texts, four applied ML not based on radiomics, five focused only on analyzing factors without developing ML models, and four assessed postoperative prediction of LNM. Ultimately, 22 studies were included (**Figure 1**) (13–34).

### 3.2 Study characteristics

Among the 22 eligible studies, seven (31.8%) were conducted in China, two (9.1%) in Italy, four (18.1%) in Germany, two (9.1%) in Sweden, three (13.6%) in the Netherlands, one (4.5%) in Israel, one (4.5%) in Switzerland, one (4.5%) in France, and one (4.5%) in Turkey. These studies were published primarily between 2017 and 2025. One study was a prospective cohort study, while the remaining 21 were case–control studies. A total of 13 studies employed radiomics based on PET/CT, and nine studies employed radiomics based on MRI (**Table 1**). A total of at least 6,301 participants were encompassed, among whom at least 1,322 had LNM. There were 14 single-center studies, six were multicenter, and two utilized databases (**Table 1**).

**Table 1:** Basic characteristics of eligible studies.

First author	Year of publication	Country	Study design	Patient source	Image source	Segmentation method	Number of cases with lymph node metastasis	Total number of cases	Number of cases in the training set	Method of generating the validation set	Number of cases in the validation set	Type of model used
Fatma Ezgi Öğülmüş (34)	2025	Turkey	Case–control	Single center	^68^Ga-PSMA PET/CT	Manual segmentation	67	229	181	Random sampling	48	DL
Vincent Bourbonne (33)	2021	France	Case–control	Single center	MRI	Manual segmentation	52	280	168	Random sampling	112	ML (ANN)
Qiaoke Ma, Bei Chen (32)	2025	China	Case–control	Single center	[^68^Ga] Ga-PSMA-617 PET/CT	Manual segmentation	45	116	82	Cross-validation	34	ML (SVM)
Chunxing Li, Jisu Hu (31)	2024	China	Case–control	Multi-center	Biparametric MRI	Automatic segmentation	126	394	263	Internal validation + external validation	74 internal validation + 57 external validation	ML (LASSON)
Snir Dekalo (30)	2024	Israel	Case–control	Database	^68^Ga-PSMA (PET/CT)		31	413	295	Internal validation	118	ML (LR)
Urs J. Muehlematter (29)	2023	Switzerland	Case–control	Multi-center	[^68^Ga]Ga-PSMA-11		77	263	173	Cross-validation + external validation	90	ML (MLR)
Giorgio Gandaglia (28)	2023	Italy	Case–control	Multi-center	PSMA PET CT		53	458		Leave-one-out cross-validation		ML (LR)
Yinzhao Wang MD (27)	2022	China	Case–control	Single center	^68^Ga‐PSMA‐PET/CT	Manual segmentation	22	66		Internal validation		ML (LR)
Xiang Liu (26)	2022	China	Case–control	Single center	mpMRI	Automatic segmentation	Image 235	Image 1116;68	Image 908	Internal validation	Image 208	ML (MLR)
Xiang Liu (25)	2021	China	Case–control	Single center	mpMRI	Manual segmentation	Image 1560;40	Image 9497;393	Image8134;309	Internal validation	Image 1258;77	DL
Oscar A. Debats (24)	2019	The Netherlands	Case–control	Single center	MRI	Manual segmentation						DL
Jan C. Peeken (23)	2020	Germany	Case–control	Single center	^68^Ga-PSMA-11-PET/CT	Manual segmentation	110	149	87	Cross-validation	62	ML (LASSON)
Elin Trägårdh (22)	2022	Sweden	Case–control	Multi-center	[^18^F]-PSMA-1007 PET-CT	Manual segmentation		120				DL
Elin Trägårdh (21)	2022	Sweden	Case–control	Single center	[^18^F]DCFPyL PET-CT	Manual segmentation		211		Internal validation		DL
Zhaonan Sun (20)	2025	China	Case–control	Multi-center	mpMRI	Manual segmentation	323	Image 28947; 1552	Image 25335	Internal validation + external validation	Image 3612; 401	DL
Suryadipto Sarkar (19)	2024	Germany	Case–control	Database	MRI	Automatic segmentation	44+			Cross-validation	88	ML (CNN)
Domiziana Santucci (18)	2024	Italy	Case–control	Single center	mpMRI		30	95		Cross-validation		
Wietske I. Luining (17)	2023	The Netherlands	Case–control	Multi-center	^18^F-DCFPyL PET/CT	Automatic segmentation	21	123	72	Internal validation + external validation	Internal: 24; external: 27	ML (RF)
Xiang Liu (16)	2022	China	Case–control	Single center	mpMRI	Manual segmentation	253	602	474	Internal validation	128	ML (RF)
A Hartenstein (15)	2020	Germany	Case–control	Single center	^68^Ga-pSMA-positivity from CT	Manual segmentation	Image 183	549			Image 1243	DL
Frederik L. Giesel (14)	2017	Germany	Case–control	Single center	PET/CT	Manual segmentation		148				
Matthijs C.F. Cysouw (13)	2021	the Netherlands	Prospective cohort study	Single center	[^18^F]DCFPyL PET		28	72		Cross-validation	72	ML (RL)

### 3.3 Quality assessment of studies

All 22 eligible studies reported imaging protocols, performed dimensionality reduction, calculated discriminative and calibration statistics, and conducted validation. However, all studies did not test scanners, perform repeated measurements at multiple time points, evaluate and discuss biological relevance, register prospective studies in trial databases, analyze cost-effectiveness, or compare models with the gold standard. Eight studies performed multiple segmentations. The scores of the studies ranged from 9 to 15. A total of 20 studies performed multivariable analyses incorporating non-radiomic features, three studies provided cutoff analyses, and 17 demonstrated potential clinical utility. Four studies provided publicly available code and data. The distribution of study scores was as follows: nine points were given for one study, 10 points for four studies, 11 points for six studies, 12 points for five studies, 13 points for three studies, and 15 points for three studies. The mean score of the studies was 11.8.

### 3.4 Meta-analysis

#### 3.4.1 Training set

**Figure 2:**
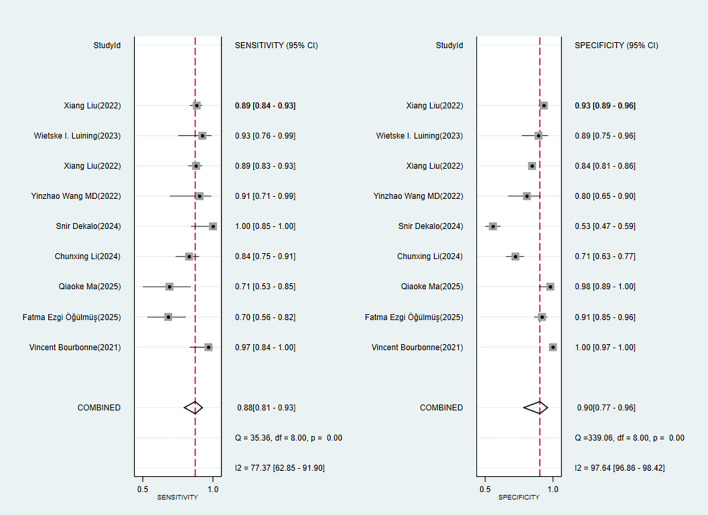
Forest plot of SEN and SPC for radiomics-based ML detection of LNM in PC.

**Figure 3:**
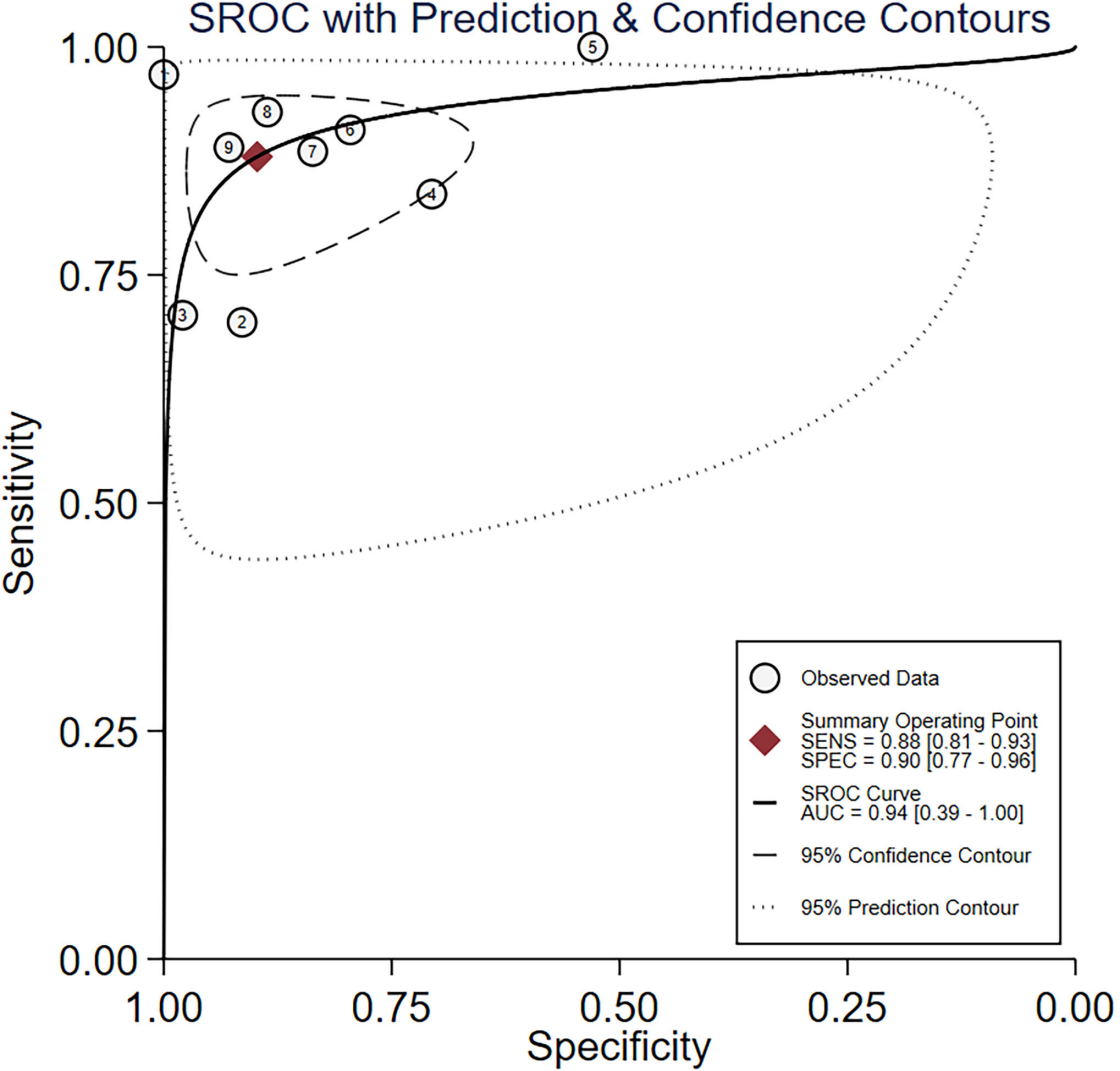
SROC for radiomics-based ML detection of LNM in PC.

**Figure 4:**
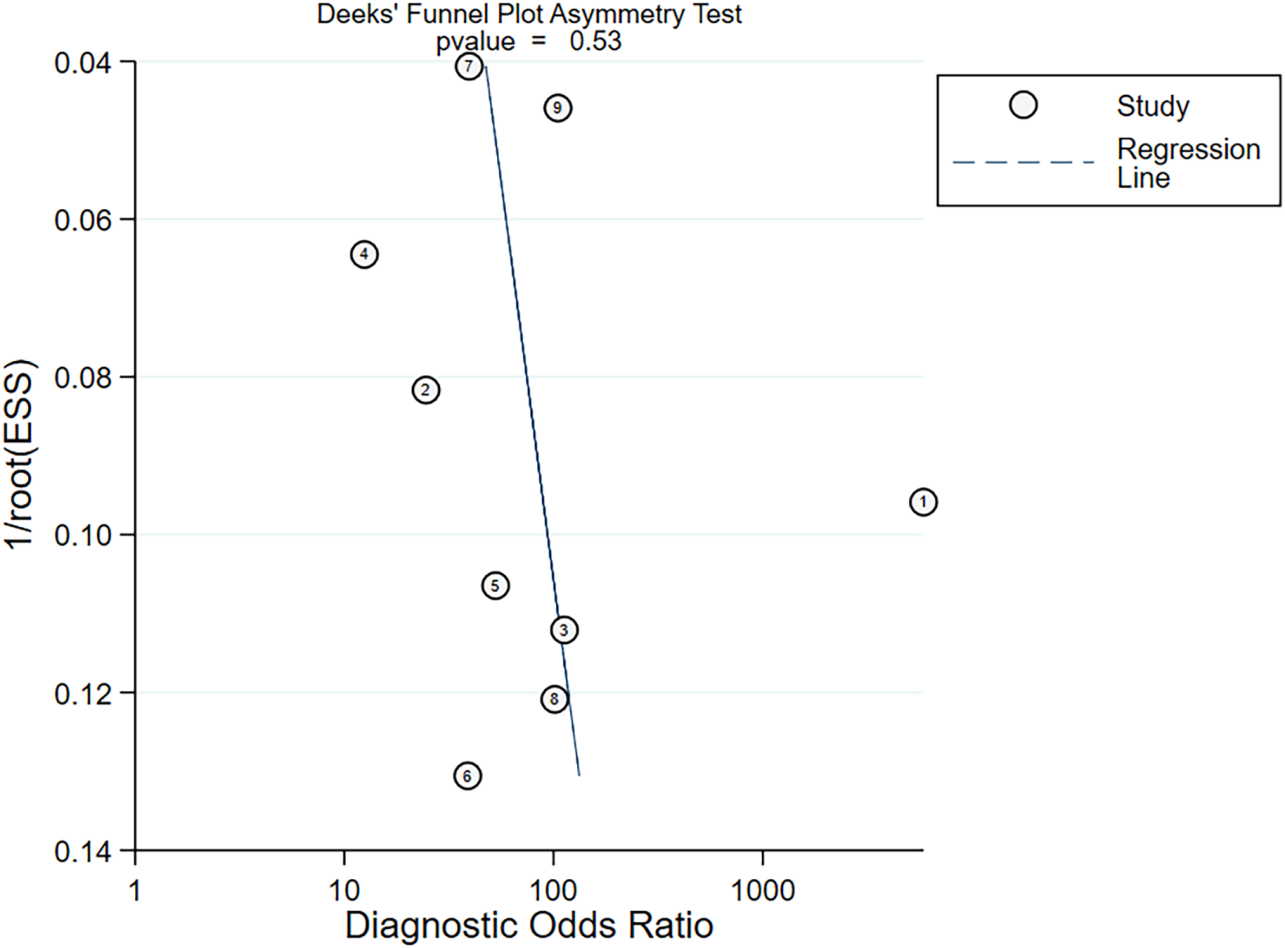
Deeks’ funnel plot assessing publication bias.

Nine studies provided diagnostic 2 × 2 tables for radiomics-based ML models in assessing LNM in PC in the training set. There was significant heterogeneity among the studies (*I*^2^ = 93%). The pooled SEN, SPC, PLR, NLR, DOR, and SROC were 0.88 (95% confidence interval (CI): 0.81–0.93), 0.90 (95% CI: 0.77–0.96), 8.6 (95% CI: 3.7–20.1), 0.13 (95% CI: 0.08–0.22), 64 (95% CI: 23–179), and 0.94 (95% CI: 0.39–1.00) (**Figures 2**, **3**). Deeks’ funnel plot did not show a marked publication bias in the training set (*p* = 0.53) (**Figure 4**).

Subgroup analyses by imaging sources were performed in the training set. In the training set, four studies reported diagnostic 2 × 2 tables for MRI-based ML models in detecting LNM in PC patients. Significant heterogeneity was noted among the studies (*I*^2^ = 80%). The pooled SEN, SPC, PLR, NLR, DOR, and SROC were 0.90 (95% CI:0.83–0.95), 0.94 (95% CI: 0.66–0.99), 14.6 (95% CI: 2.0–106.9), 0.10 (95% CI:0.05–0.21), 140 (95% CI: 10–1,945), and 0.95 (95% CI: 0.55–1.00) (**Supplementary Figures S1**, **S2**). The publication bias was insignificant (*p* = 0.43) (**Supplementary Figure S3**).

In the training set, five studies reported diagnostic 2 × 2 tables for ML models based on PET/CT. There was significant heterogeneity among the studies (*I*^2^ = 96%). The pooled SEN, SPC, PLR, NLR, DOR, and SROC were 0.89 (95% CI:0.69–0.97), 0.85 (95% CI: 0.69–0.93), 6.0 (95% CI: 3.0–11.8), 0.13 (95% CI:0.04–0.38), 47 (95% CI: 20–110), and 0.93 (95% CI: 0.63–0.99) (**Supplementary Figures S4**, **S5**). Deeks’ funnel plot indicated an insignificant publication bias (*p*= 0.20) (**Supplementary Figure S6**).

#### 3.4.2 Validation set

**Figure 5:**
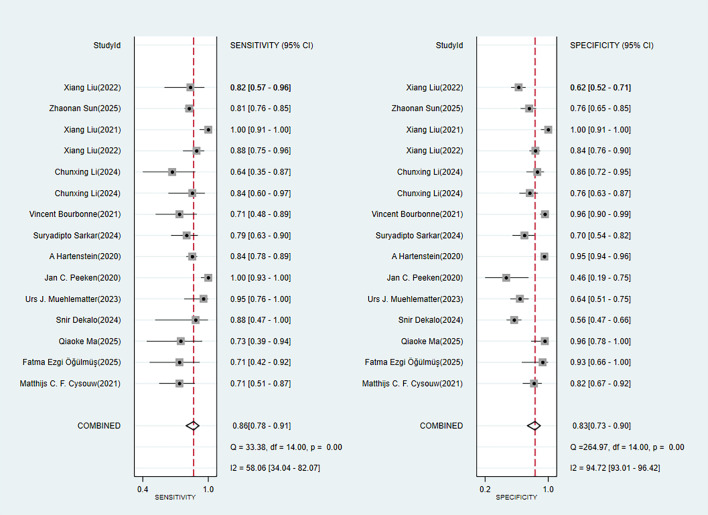
Forest plot of SEN and SPC for radiomics-based ML.

**Figure 6:**
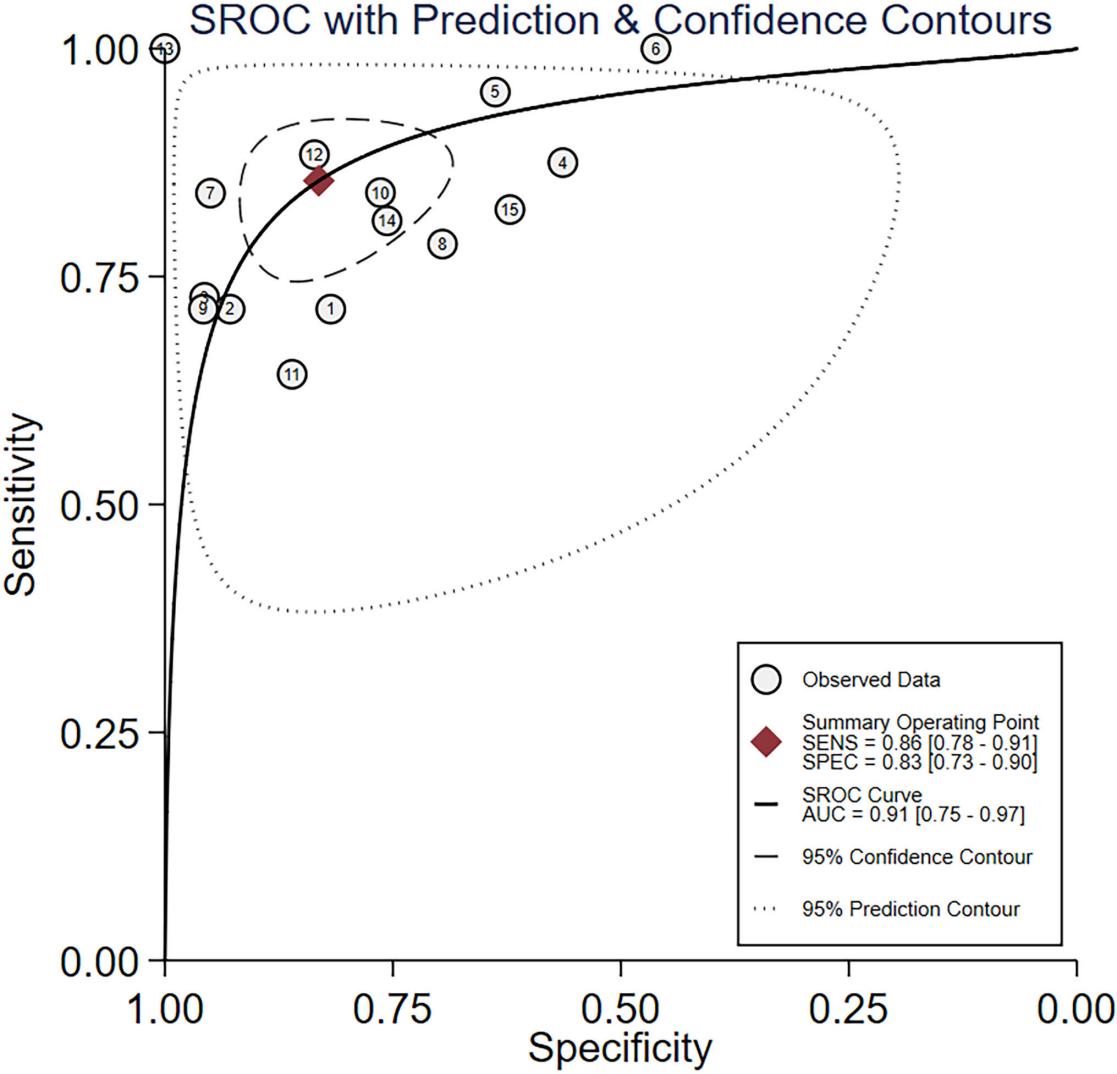
SROC of radiomics-based ML.

**Figure 7:**
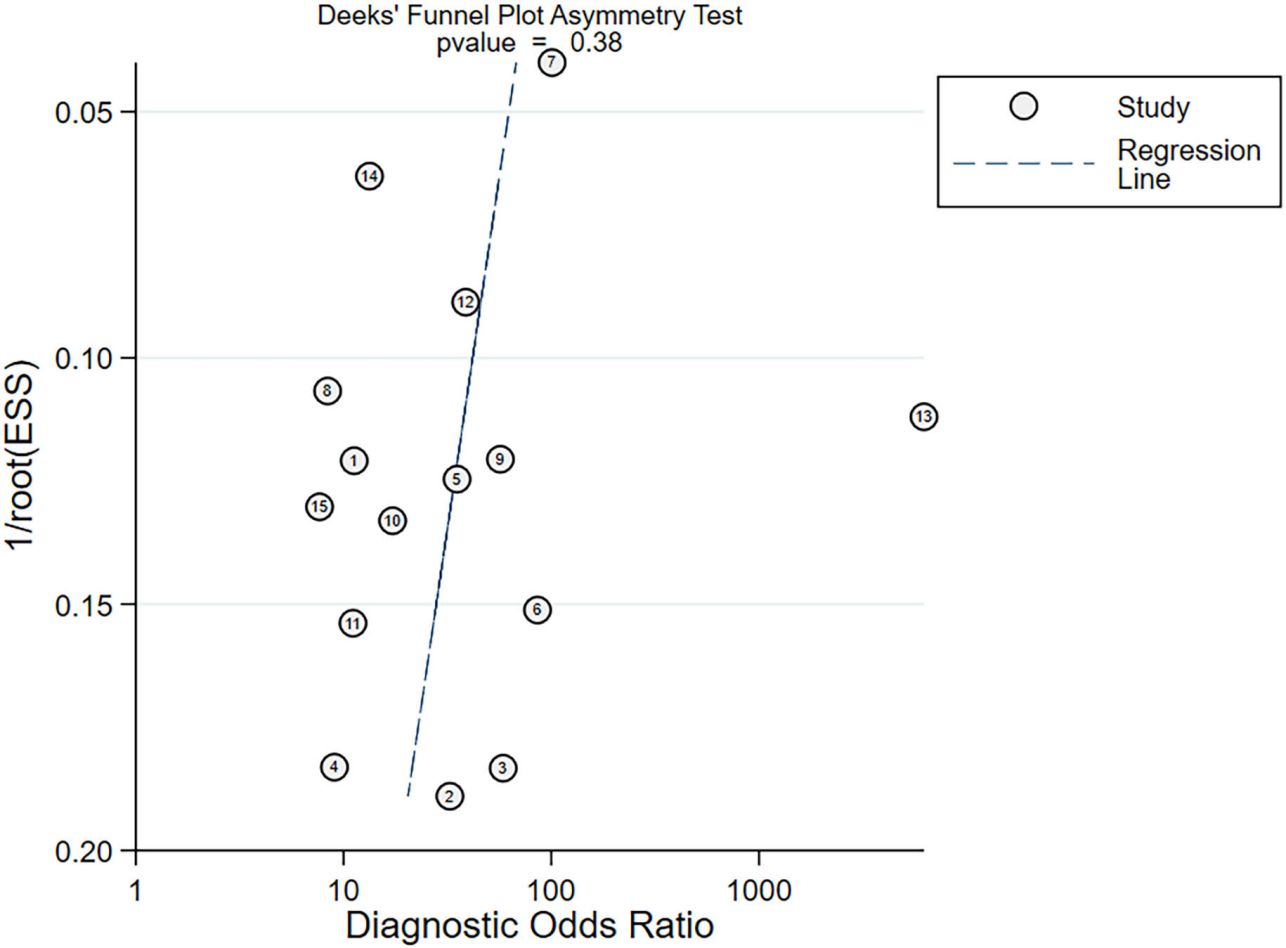
Deeks’ funnel plot of radiomics-based ML.

A total of 14 studies provided diagnostic 2 × 2 tables for radiomics-based ML models in detecting LNM in PC in the validation set. There was significant heterogeneity among the studies (*I*^2^ = 96%). The pooled SEN, SPC, PLR, NLR, DOR, and SROC were 0.86 (95% CI: 0.78–0.91), 0.83 (95% CI: 0.73–0.90), 5.1 (95% CI: 3.2–8.1), 0.17 (95% CI: 0.11–0.26), 29 (95% CI: 15–57), and 0.91 (95% CI: 0.75–0.97) (**Figures 5**, **6**). Deeks’ funnel plot showed no significant publication bias (*p* = 0.38) (**Figure 7**).

Subgroup analyses by imaging sources were performed in the validation set. Seven studies provided 2 × 2 tables for MRI-based ML. There was relatively high heterogeneity among the studies (*I*^2^ = 61%). The pooled SEN, SPC, PLR, NLR, DOR, and SROC were 0.84 (95% CI:0.78–0.89), 0.86 (95% CI: 0.71–0.94), 5.9 (95% CI: 2.6–13.5), 0.18 (95% CI:0.11–0.29), 32 (95% CI: 9–113), and 0.90 (95% CI: 0.63–0.98) (**Supplementary Figures S7**, **S8**). The publication bias was not significant (*p* = 0.70) (**Supplementary Figure S9**).

In the validation set, seven studies reported diagnostic 2 × 2 tables for PET/CT-based ML in detecting LNM in PC patients. Significant heterogeneity was observed among the studies (*I*^2^ = 90%). The pooled SEN, SPC, PLR, NLR, DOR, and SROC were 0.89 (95% CI:0.75–0.96), 0.82 (95% CI: 0.63–0.93), 5.0 (95% CI: 2.3–10.6), 0.13 (95% CI:0.06–0.29), 37 (95% CI: 16–87), and 0.93 (95% CI: 0.77–0.98) (**Supplementary Figures S10**, **S11**). No publication bias was noted (*p* = 0.06) (**Supplementary Figure S12**).

Subgroup analyses by model types were performed. A total of 10 studies on traditional ML provided diagnostic 2 × 2 tables. There was significant heterogeneity among the studies (*I*^2^ = 94%). The pooled SEN, SPC, PLR, NLR, DOR, and SROC were 0.85 (95% CI:0.76–0.91), 0.77 (95% CI: 0.66–0.86), 3.7 (95% CI: 2.5–5.5), 0.20 (95% CI:0.13–0.30), 19 (95% CI: 12–31), and 0.89 (95% CI: 0.72–0.96) (**Supplementary Figures S13**, **S14**). Deeks’ funnel plot suggested no publication bias (*p* = 0.88) (**Supplementary Figure S15**).

Four studies on DL models reported diagnostic 2 × 2 tables. There was significant heterogeneity among the studies (*I*^2^ = 80%). The pooled SEN, SPC, PLR, NLR, DOR, and SROC were 0.88 (95% CI:0.75–0.95), 0.97 (95% CI: 0.58–1.00), 31.2 (95% CI: 1.3–761.7), 0.12 (95% CI:0.05–0.29), 258 (95% CI: 5–14,123), and 0.95 (95% CI: 0.19–1.00) (**Supplementary Figures S16**, **S17**). The publication bias was insignificant (*p* = 0.77) (**Supplementary Figure S18**).

## 4 Discussion

### 4.1 Summary of the main findings

In our study, radiomics-based ML appeared to be an effective approach to assisting in the diagnosis of LNM in PC. The pooled results indicated that MRI-based models achieved a SEN of 0.84 (95% CI: 0.78–0.89) and SPC of 0.86 (95% CI: 0.71–0.94), while PET/CT-based models showed a SEN of 0.89 (95% CI: 0.75–0.96) and SPC of 0.82 (95% CI: 0.63–0.93). These models exhibited good performance in the prediction of LNM. The SEN and SPC of DL models were 0.88 (95% CI: 0.75–0.95) and 0.97 (95% CI: 0.58–1.00), respectively, suggesting a potentially higher diagnostic accuracy than traditional ML models.

### 4.2 Comparison with previous reviews

Prior studies have examined the performance of ML in predicting LNM in PC. Wang et al. (35) have conducted a systematic review comparing clinical and radiomic features for predicting LNM. Their review reports that radiomic features are more accurate, with a pooled SEN of 0.81 (95% CI: 0.67–0.89) and SPC of 0.82 (95% CI: 0.75–0.88). Similarly, Zheng et al. (36) have reported a pooled SEN of 0.81 (95% CI: 0.62–0.91) and SPC of 0.83 (95% CI: 0.73–0.90) for radiomics-based ML. However, these prior studies did not separately analyze radiomics sources, encompassed limited radiomics studies, and lacked detailed discussion on imaging sources and modeling approaches. Building upon these studies, our study specifically analyzed radiomics-based models for diagnosing LNM and performed subgroup analyses by imaging sources and model types, highlighting the potential superior accuracy of DL models.

### 4.3 Image selection in the modeling process

In the included studies, radiomics for detecting prostate LNM was primarily sourced from MRI or PET/CT, both of which are clinically relevant. Nevertheless, preoperative imaging-based assessment of LNM remains limited. Over 65% of patients scheduled for extended pelvic lymph node dissection (ePLND) are pathologically node-negative (37). The mpMRI detection of lymph nodes depends on size, yielding low SEN. The meta-analysis results indicate that PSMA PET/CT demonstrates a SEN of 0.74 (95% CI: 0.62–0.85) and a SPC of 0.96 (95% CI: 0.93–0.98). mpMRI exhibits a lower SEN of 0.45 (95% CI: 0.32–0.57) and a SPC of 0.92 (95% CI: 0.86–0.97). Conventional mpMRI has limitations, but combining diffusion-weighted imaging (DWI) with high-resolution 3D T2-weighted morphological imaging improves the detection accuracy (38). Although PSMA PET/CT is more accurate, it incurs higher costs. In our study, the difference in the diagnostic performance of LNM was insignificant between models based on MRI and PET/CT, supporting the use of cost-effective MRI.

### 4.4 Model selection in the modeling process

Few studies employed DL models to predict LNM. DL, a generative neural network, integrates image segmentation, feature extraction, and texture selection for training, retaining more image information, and improving model performance (39). In our validation set, the DL models outperformed the traditional ML models. Traditional ML has several limitations in segmentation, texture extraction, and feature selection—for instance, image segmentation heavily depends on operator experience, introducing inter-observer variability (40). Different software (e.g., 3D Slicer, ITK-SNAP) produces heterogeneous texture features (41, 42). Feature selection reduces hundreds to thousands of features down to a few (often <20), resulting in information loss. Segmentation and feature selection also require significant labor. These limitations suggest that DL possibly presents a more automated and accurate method for image processing (43). Overall, DL appears to be more accurate and may be used to develop intelligent diagnostic tools.

High heterogeneity was observed in our study. This heterogeneity primarily stems from several factors. First, substantial differences in image protocols contribute to potential heterogeneity. The original studies did not discuss the impact of these different image protocols on imaging. Second, image segmentation methods are diverse, primarily manual, and automated, which rely heavily on the experience of the operators. While a few studies have discussed heterogeneity in segmentation methods, numerous studies fail to address it, creating a potential source of heterogeneity. Third, the predictive performance of different ML methods may vary. Fourth, different clinical features may also increase potential heterogeneity. Future research should further standardize radiomics implementation procedures to objectively and accurately reflect the diagnostic performance of radiomics for diseases, thereby minimizing heterogeneity.

The average RQS of the included studies was only 11 points. This score is generally low and reflects the widespread deficiencies in methodologies in current radiomics research. This low research quality poses a significant challenge to the evaluation of the true performance of models. Specifically, a low RQS often indicates that most studies do not perform external validation, test the robustness of features, or adequately implement feature selection strategies to prevent overfitting. This limitation can easily lead to optimism bias, that is, the published predictive power (such as the SROC value) may be far higher than its performance in the real world, failing to represent the generalizability of models. Furthermore, in terms of clinical applicability, the low RQS score reveals a gap in the clinical translation path of current research. Most studies do not adopt prospective designs, analyze cost-effectiveness, provide decision curves, or offer publicly available source code or imaging data. This black box research method not only reduces the reproducibility of evidence but also makes it difficult for clinicians to assess the stability of models under different equipment parameters and scanning protocols, thus severely limiting the practical application of radiomics models in clinical auxiliary diagnosis. Therefore, although our results show high diagnostic performance of ML models, caution should be exercised when interpreting these results, given the overall low quality of evidence.

The validation set is a subset of the original training data specifically used to evaluate model performance, perform hyperparameter tuning, and select models during training. It directly impacts the effectiveness of model tuning and the objective evaluation of model performance. External validation, on the other hand, is a standard method for evaluating the generalizability and clinical application of a model using entirely new, independent data. Our analysis included 22 original studies, but only four of them conducted external validation. Because this study used a bivariate mixed-effects model, a sufficient number of studies were needed for quantitative analysis. Therefore, given the limited number of studies, we did not further conduct subgroup analysis by internal and external validation.

### 4.5 Challenges of DL

Despite its potential in developing diagnostic assistance tools, DL faces several challenges. First, variations in imaging protocols can affect model stability. Differences in image quality across protocols possibly alter lesion appearance, which can substantially impact automated segmentation and, consequently, compromise the stability of DL models (44, 45). Second, DL relies on complex neural network architectures that theoretically require large datasets to ensure stable training. This is because when neural networks estimate the sample size, one neuron corresponds to 10 samples or 10 images. Therefore, complex neural networks contain a large number of neurons. Measures such as transfer learning or federated learning may partially resolve this problem. However, most current studies employ only small numbers of images, which imposes significant limitations on the interpretation of model stability (46, 47). Third, adequate validation of constructed models is essential because variations in imaging parameters can lead to substantial differences in image characteristics.

Therefore, models based on imaging data, whether DL or traditional ML, need to be externally validated to assess their generalizability. Most existing studies rely on simple random sampling or cross-validation, while studies on external validation are scarce. Future research should incorporate multi-center datasets and evaluate constructed models more comprehensively to develop or update broadly applicable models (48, 49).

### 4.6 Hardware, processes, and ethics required for models

To promote and deploy radiomics in clinical practice, we first need to prepare image segmentation software, including common manual segmentation software such as 3D Slice, ITK-SNAP, EISeg, and Labelme. Some commercially available automated segmentation software can also be used to label lesions in images. Then, clinical features and information such as color, texture, shape, and size extracted from images can be combined to build a better-performing ML model. However, this approach incurs costs, particularly time costs during image segmentation, and may cause information loss. This is because the feature selection process requires filtering a small number of features from a large pool of texture features. Therefore, in clinical deployment, more intelligent DL is recommended since DL can use images generated by segmentation software to train models. After fully validating the model performance, it may assist in the intelligent diagnosis of LNM. However, it is necessary to overcome the ethical challenges involved in this process, especially protecting the critical information of patients.

### 4.7 Strengths and limitations

This study systematically evaluated the diagnostic performance of radiomics for detecting LNM in PC. Furthermore, subgroup analyses were performed by different imaging modalities and model types. Nevertheless, several limitations should be considered. First, despite systematic literature retrieval, the number of eligible studies is limited, restricting in-depth discussion on modeling approaches, imaging modalities, and validation strategies. In particular, multi-center external validation in different regions is lacking, which limits the use of predictive models. Second, the eligible studies employed diverse imaging protocols. Hence, evaluating how protocol variations influence model performance is difficult. Third, although DL demonstrated superior accuracy, the number of related studies is small, and there is limited discussion regarding segmentation types and lesion localization. Fourth, none of the eligible studies directly compared model performance with clinical experts. Hence, it is infeasible to compare the accuracy of models and experts. Fifth, the RQS scores indicated that the quality of the original studies was concerning. The included studies are difficult to score highly in several items. Firstly, prospective registration is required. However, the included studies did not perform prospective registration, resulting in a seven-point loss. Secondly, multicenter validation is lacking, which also contributes significantly to the loss of points. Hence, the quality of the included studies is overall low, and thus our analysis results should be interpreted with caution. Sixth, because this study employed a bivariate mixed-effects model, a sufficient number of studies are needed for quantitative analysis. However, only four original studies performed external validation (two studies on MRI and two studies on PET/CT). Given the limited number of studies available, subgroup analysis by internal and external validation of models is not performed.

## 5 Conclusions

Radiomics shows promising diagnostic performance for detecting LNM in PC. DL models are more accurate than traditional ML models. However, before widespread clinical implementation, several challenges must be addressed. Nonetheless, given the limited sample sizes, insufficient external validation, and heterogeneity in imaging protocols, future research should include more multi-center images from different regions. Meanwhile, it is necessary to standardize the imaging and segmentation protocols to improve transparency and reduce heterogeneity, thereby constructing more widely applicable and high-performance radiomics-based ML models to improve the accuracy of early detection of LNM in PC.

## Data Availability

The original contributions presented in the study are included in the article/**Supplementary Material**. Further inquiries can be directed to the corresponding author.
