# Supplementary material for: The diagnostic value of radiomics-based machine learning for lymph node metastasis in prostate cancer: a systematic review and meta-analysis
Source: Front Oncol. 2026 Feb 12;16:1710716. doi: 10.3389/fonc.2026.1710716 (PMC12935596; doi:10.3389/fonc.2026.1710716)
Supplement: Supplementary file 1 [file DataSheet1.docx]

**Table S1** Literature search strategy

**1.Pubmed**

| Search number | Query | Results |
| --- | --- | --- |
| #1 | "Prostatic Neoplasms"[Mesh] | 159212 |
| #2 | (((((((((((((((((((((Prostatic Neoplasms[Title/Abstract]) OR (Prostate Neoplasms[Title/Abstract])) OR (Prostate Neoplasm[Title/Abstract])) OR (Prostatic Neoplasm[Title/Abstract])) OR (Prostate Cancer[Title/Abstract])) OR (Prostate Cancers[Title/Abstract])) OR (Prostatic Cancer[Title/Abstract])) OR (Prostatic Cancers[Title/Abstract])) OR (prostate tumor[Title/Abstract])) OR (prostate gland tumor[Title/Abstract])) OR (prostate gland tumour[Title/Abstract])) OR (prostate neoplasia[Title/Abstract])) OR (prostate neoplasm[Title/Abstract])) OR (prostate tumour[Title/Abstract])) OR (prostatic neoplasia[Title/Abstract])) OR (prostatic tumor[Title/Abstract])) OR (prostatic tumour[Title/Abstract])) OR (prostate gland cancer[Title/Abstract])) OR (prostate malignancy[Title/Abstract])) OR (prostate malignant neoplasm[Title/Abstract])) OR (prostatic cancer[Title/Abstract])) OR (prostatic malignancy[Title/Abstract]) | 169639 |
| #3 | ("Prostatic Neoplasms"[Mesh]) OR ((((((((((((((((((((((Prostatic Neoplasms[Title/Abstract]) OR (Prostate Neoplasms[Title/Abstract])) OR (Prostate Neoplasm[Title/Abstract])) OR (Prostatic Neoplasm[Title/Abstract])) OR (Prostate Cancer[Title/Abstract])) OR (Prostate Cancers[Title/Abstract])) OR (Prostatic Cancer[Title/Abstract])) OR (Prostatic Cancers[Title/Abstract])) OR (prostate tumor[Title/Abstract])) OR (prostate gland tumor[Title/Abstract])) OR (prostate gland tumour[Title/Abstract])) OR (prostate neoplasia[Title/Abstract])) OR (prostate neoplasm[Title/Abstract])) OR (prostate tumour[Title/Abstract])) OR (prostatic neoplasia[Title/Abstract])) OR (prostatic tumor[Title/Abstract])) OR (prostatic tumour[Title/Abstract])) OR (prostate gland cancer[Title/Abstract])) OR (prostate malignancy[Title/Abstract])) OR (prostate malignant neoplasm[Title/Abstract])) OR (prostatic cancer[Title/Abstract])) OR (prostatic malignancy[Title/Abstract])) | 205744 |
| #4 | Radiomics[MeSH Terms] | 2275 |
| #5 | ((((((((((((((((((((((((((((((((Radiomics[Title/Abstract]) OR (Radiomic[Title/Abstract])) OR (machine learning[Title/Abstract])) OR (artificial intelligence[Title/Abstract])) OR (Transfer Learning[Title/Abstract])) OR (prediction model[Title/Abstract])) OR (Deep learning[Title/Abstract])) OR (ResNet[Title/Abstract])) OR (AlexNet[Title/Abstract])) OR (VGGNet[Title/Abstract])) OR (GoogLeNet[Title/Abstract])) OR (Ensemble Learning[Title/Abstract])) OR (risk model[Title/Abstract])) OR (risk score[Title/Abstract])) OR (random forest[Title/Abstract])) OR (neural network[Title/Abstract])) OR (neural networks[Title/Abstract])) OR (CNN[Title/Abstract])) OR (K-Nearest Neighbor[Title/Abstract])) OR (Support vector machine[Title/Abstract])) OR (SVM[Title/Abstract])) OR (Gradient Boosting Machine[Title/Abstract])) OR (Nomogram[Title/Abstract])) OR (XGBoost[Title/Abstract])) OR (Adaboost[Title/Abstract])) OR (LightGBM[Title/Abstract])) OR (CatBoost[Title/Abstract])) OR (Gradient Boosting[Title/Abstract])) OR (Decision tree[Title/Abstract])) OR (Regression Trees[Title/Abstract])) OR (Naive Bayesian[Title/Abstract])) OR (Multilayer perceptron[Title/Abstract])) OR (Bayesian network[Title/Abstract]) | 477918 |
| #6 | (Radiomics[MeSH Terms]) OR (((((((((((((((((((((((((((((((((Radiomics[Title/Abstract]) OR (Radiomic[Title/Abstract])) OR (machine learning[Title/Abstract])) OR (artificial intelligence[Title/Abstract])) OR (Transfer Learning[Title/Abstract])) OR (prediction model[Title/Abstract])) OR (Deep learning[Title/Abstract])) OR (ResNet[Title/Abstract])) OR (AlexNet[Title/Abstract])) OR (VGGNet[Title/Abstract])) OR (GoogLeNet[Title/Abstract])) OR (Ensemble Learning[Title/Abstract])) OR (risk model[Title/Abstract])) OR (risk score[Title/Abstract])) OR (random forest[Title/Abstract])) OR (neural network[Title/Abstract])) OR (neural networks[Title/Abstract])) OR (CNN[Title/Abstract])) OR (K-Nearest Neighbor[Title/Abstract])) OR (Support vector machine[Title/Abstract])) OR (SVM[Title/Abstract])) OR (Gradient Boosting Machine[Title/Abstract])) OR (Nomogram[Title/Abstract])) OR (XGBoost[Title/Abstract])) OR (Adaboost[Title/Abstract])) OR (LightGBM[Title/Abstract])) OR (CatBoost[Title/Abstract])) OR (Gradient Boosting[Title/Abstract])) OR (Decision tree[Title/Abstract])) OR (Regression Trees[Title/Abstract])) OR (Naive Bayesian[Title/Abstract])) OR (Multilayer perceptron[Title/Abstract])) OR (Bayesian network[Title/Abstract])) | 477919 |
| #7 | Lymphatic Metastasis[MeSH Terms] | 101653 |
| #8 | (((((((((((Lymphatic Metastasis[Title/Abstract]) OR (Lymphatic Metastases[Title/Abstract])) OR (Lymph Node Metastasis[Title/Abstract])) OR (Lymph Node Metastases[Title/Abstract])) OR (Lymph node invasion[Title/Abstract])) OR (Lymph node involvement[Title/Abstract])) OR (lymph gland metastases[Title/Abstract])) OR (lymph gland metastasis[Title/Abstract])) OR (lymph metastases[Title/Abstract])) OR (lymph metastasis[Title/Abstract])) OR (lymph node positivity[Title/Abstract])) OR (lymphoid metastasis[Title/Abstract]) | 83872 |
| #9 | (Lymphatic Metastasis[MeSH Terms]) OR ((((((((((((Lymphatic Metastasis[Title/Abstract]) OR (Lymphatic Metastases[Title/Abstract])) OR (Lymph Node Metastasis[Title/Abstract])) OR (Lymph Node Metastases[Title/Abstract])) OR (Lymph node invasion[Title/Abstract])) OR (Lymph node involvement[Title/Abstract])) OR (lymph gland metastases[Title/Abstract])) OR (lymph gland metastasis[Title/Abstract])) OR (lymph metastases[Title/Abstract])) OR (lymph metastasis[Title/Abstract])) OR (lymph node positivity[Title/Abstract])) OR (lymphoid metastasis[Title/Abstract])) | 147525 |
| #10 | ((("Prostatic Neoplasms"[Mesh]) OR ((((((((((((((((((((((Prostatic Neoplasms[Title/Abstract]) OR (Prostate Neoplasms[Title/Abstract])) OR (Prostate Neoplasm[Title/Abstract])) OR (Prostatic Neoplasm[Title/Abstract])) OR (Prostate Cancer[Title/Abstract])) OR (Prostate Cancers[Title/Abstract])) OR (Prostatic Cancer[Title/Abstract])) OR (Prostatic Cancers[Title/Abstract])) OR (prostate tumor[Title/Abstract])) OR (prostate gland tumor[Title/Abstract])) OR (prostate gland tumour[Title/Abstract])) OR (prostate neoplasia[Title/Abstract])) OR (prostate neoplasm[Title/Abstract])) OR (prostate tumour[Title/Abstract])) OR (prostatic neoplasia[Title/Abstract])) OR (prostatic tumor[Title/Abstract])) OR (prostatic tumour[Title/Abstract])) OR (prostate gland cancer[Title/Abstract])) OR (prostate malignancy[Title/Abstract])) OR (prostate malignant neoplasm[Title/Abstract])) OR (prostatic cancer[Title/Abstract])) OR (prostatic malignancy[Title/Abstract]))) AND ((Radiomics[MeSH Terms]) OR (((((((((((((((((((((((((((((((((Radiomics[Title/Abstract]) OR (Radiomic[Title/Abstract])) OR (machine learning[Title/Abstract])) OR (artificial intelligence[Title/Abstract])) OR (Transfer Learning[Title/Abstract])) OR (prediction model[Title/Abstract])) OR (Deep learning[Title/Abstract])) OR (ResNet[Title/Abstract])) OR (AlexNet[Title/Abstract])) OR (VGGNet[Title/Abstract])) OR (GoogLeNet[Title/Abstract])) OR (Ensemble Learning[Title/Abstract])) OR (risk model[Title/Abstract])) OR (risk score[Title/Abstract])) OR (random forest[Title/Abstract])) OR (neural network[Title/Abstract])) OR (neural networks[Title/Abstract])) OR (CNN[Title/Abstract])) OR (K-Nearest Neighbor[Title/Abstract])) OR (Support vector machine[Title/Abstract])) OR (SVM[Title/Abstract])) OR (Gradient Boosting Machine[Title/Abstract])) OR (Nomogram[Title/Abstract])) OR (XGBoost[Title/Abstract])) OR (Adaboost[Title/Abstract])) OR (LightGBM[Title/Abstract])) OR (CatBoost[Title/Abstract])) OR (Gradient Boosting[Title/Abstract])) OR (Decision tree[Title/Abstract])) OR (Regression Trees[Title/Abstract])) OR (Naive Bayesian[Title/Abstract])) OR (Multilayer perceptron[Title/Abstract])) OR (Bayesian network[Title/Abstract])))) AND ((Lymphatic Metastasis[MeSH Terms]) OR ((((((((((((Lymphatic Metastasis[Title/Abstract]) OR (Lymphatic Metastases[Title/Abstract])) OR (Lymph Node Metastasis[Title/Abstract])) OR (Lymph Node Metastases[Title/Abstract])) OR (Lymph node invasion[Title/Abstract])) OR (Lymph node involvement[Title/Abstract])) OR (lymph gland metastases[Title/Abstract])) OR (lymph gland metastasis[Title/Abstract])) OR (lymph metastases[Title/Abstract])) OR (lymph metastasis[Title/Abstract])) OR (lymph node positivity[Title/Abstract])) OR (lymphoid metastasis[Title/Abstract]))) | 318 |

**2.Cochrane**

| Search number | Query | Results |
| --- | --- | --- |
| #1 | MeSH descriptor: [Prostatic Neoplasms] explode all trees | 9071 |
| #2 | (Prostatic Neoplasms):ti,ab,kw OR (Prostate Neoplasms):ti,ab,kw OR (Prostate Neoplasm):ti,ab,kw OR (Prostatic Neoplasm):ti,ab,kw OR (Prostate Cancer):ti,ab,kw | 19403 |
| #3 | (Prostate Cancers):ti,ab,kw OR (Prostatic Cancer):ti,ab,kw OR (Prostatic Cancers):ti,ab,kw OR (prostate tumor):ti,ab,kw OR (prostate gland tumor):ti,ab,kw | 11782 |
| #4 | (prostate gland tumour):ti,ab,kw OR (prostate neoplasia):ti,ab,kw OR (prostate neoplasm):ti,ab,kw OR (prostate tumour):ti,ab,kw OR (prostatic neoplasia):ti,ab,kw | 6088 |
| #5 | (prostatic tumor):ti,ab,kw OR (prostatic tumour):ti,ab,kw OR (prostate gland cancer):ti,ab,kw OR (prostate malignancy):ti,ab,kw OR (prostate malignant neoplasm):ti,ab,kw | 3272 |
| #6 | (prostatic cancer):ti,ab,kw OR (prostatic malignancy):ti,ab,kw | 9134 |
| #7 | #1 or #2 or #3 or #4 or #5 or #6 | 19662 |
| #8 | (Radiomics):ti,ab,kw OR (Radiomic):ti,ab,kw OR (machine learning):ti,ab,kw OR (artificial intelligence):ti,ab,kw OR (Transfer Learning):ti,ab,kw | 8304 |
| #9 | (prediction model):ti,ab,kw OR (Deep learning):ti,ab,kw OR (ResNet):ti,ab,kw OR (AlexNet):ti,ab,kw OR (VGGNet):ti,ab,kw | 8235 |
| #10 | (GoogLeNet):ti,ab,kw OR (Ensemble Learning):ti,ab,kw OR (risk model):ti,ab,kw OR (risk score):ti,ab,kw OR (random forest):ti,ab,kw | 71914 |
| #11 | (neural network):ti,ab,kw OR (neural networks):ti,ab,kw OR (CNN):ti,ab,kw OR (K-Nearest Neighbor):ti,ab,kw OR (Support vector machine):ti,ab,kw | 4529 |
| #12 | (SVM):ti,ab,kw OR (Gradient Boosting Machine):ti,ab,kw OR (Nomogram):ti,ab,kw OR (XGBoost):ti,ab,kw OR (Adaboost):ti,ab,kw | 2474 |
| #13 | (LightGBM):ti,ab,kw OR (CatBoost):ti,ab,kw OR (Gradient Boosting):ti,ab,kw OR (Decision tree):ti,ab,kw OR (Regression Trees):ti,ab,kw | 1375 |
| #14 | (Naive Bayesian):ti,ab,kw OR (Multilayer perceptron):ti,ab,kw OR (Bayesian network):ti,ab,kw | 584 |
| #15 | #8 or #9 or #10 or #11 or #12 or #13 or #14 | 86812 |
| #16 | (Lymphatic Metastasis):ti,ab,kw OR (Lymphatic Metastases):ti,ab,kw OR (Lymph Node Metastasis):ti,ab,kw OR (Lymph Node Metastases):ti,ab,kw OR (Lymph node invasion):ti,ab,kw | 7410 |
| #17 | (Lymph node involvement):ti,ab,kw OR (lymph gland metastases):ti,ab,kw OR (lymph gland metastasis):ti,ab,kw OR (ymph metastases):ti,ab,kw OR (lymph metastasis):ti,ab,kw | 6656 |
| #18 | (lymph node positivity):ti,ab,kw OR (lymphoid metastasis):ti,ab,kw | 270 |
| #19 | #16 or #17 or #18 | 8414 |
| #20 | #7 and #15 and #19 | 185 |

**3.Embase**

| Search number | Query | Results |
| --- | --- | --- |
| #2 | 'prostate tumor'/exp | 339600 |
| #3 | 'prostate tumor':ab,ti OR 'prostatic neoplasms':ab,ti OR 'prostate neoplasms':ab,ti OR 'prostatic neoplasm':ab,ti OR 'prostate cancer':ab,ti OR 'prostate cancers':ab,ti OR 'prostatic cancers':ab,ti OR 'prostate gland tumor':ab,ti OR 'prostate gland tumour':ab,ti OR 'prostate neoplasia':ab,ti OR 'prostate neoplasm':ab,ti OR 'prostate tumour':ab,ti OR 'prostatic neoplasia':ab,ti OR 'prostatic tumor':ab,ti OR 'prostatic tumou':ab,ti OR 'prostate gland cancer':ab,ti OR 'prostate malignancy':ab,ti OR 'prostate malignant neoplasm':ab,ti OR 'prostatic cancer':ab,ti OR 'prostatic malignancy':ab,ti | 257398 |
| #4 | #2 OR #3 | 359852 |
| #5 | 'radiomics'/exp | 15390 |
| #6 | radiomics:ab,ti OR radiomic:ab,ti OR 'machine learning':ab,ti OR 'artificial intelligence':ab,ti OR 'transfer learning':ab,ti OR 'prediction model':ab,ti OR 'deep learning':ab,ti OR resnet:ab,ti OR alexnet:ab,ti OR vggnet:ab,ti OR googlenet:ab,ti OR 'ensemble learning':ab,ti OR 'risk model':ab,ti OR 'risk score':ab,ti OR 'random forest':ab,ti OR 'neural network':ab,ti OR 'neural networks':ab,ti OR cnn:ab,ti OR 'k-nearest neighbor':ab,ti OR 'support vector machine':ab,ti OR svm:ab,ti OR 'gradient boosting machine':ab,ti OR nomogram:ab,ti OR xgboost:ab,ti OR adaboost:ab,ti OR lightgbm:ab,ti OR catboost:ab,ti OR 'gradient boosting':ab,ti OR 'decision tree':ab,ti OR 'regression trees':ab,ti OR 'naive bayesian':ab,ti OR 'multilayer perceptron':ab,ti OR 'bayesian network':ab,ti | 560258 |
| #7 | #5 OR #6 | 561191 |
| #8 | 'lymph node metastasis'/exp | 194378 |
| #9 | 'lymph node metastasis':ab,ti OR 'lymphatic metastasis':ab,ti OR 'lymphatic metastases':ab,ti OR 'lymph node metastases':ab,ti OR 'lymph node invasion':ab,ti OR 'lymph node involvement':ab,ti OR 'lymph gland metastases':ab,ti OR 'lymph gland metastasis':ab,ti OR 'ymph metastases':ab,ti OR 'lymph metastasis':ab,ti OR 'lymph node positivity':ab,ti OR 'lymphoid metastasis':ab,ti | 115532 |
| #10 | #8 OR #9 | 224260 |
| #11 | #4 AND #7 AND #10 | 709 |

**4.Web of science**

| Search number | Query | Results |
| --- | --- | --- |
| #4 | "TS=(Prostatic Neoplasms OR Prostate Neoplasms OR Prostate Neoplasm OR Prostatic Neoplasm OR Prostate Cancer OR Prostate Cancers OR Prostatic Cancer OR Prostatic Cancers OR prostate tumor OR prostate gland tumor OR prostate gland tumour OR prostate neoplasia OR prostate neoplasm OR prostate tumour OR prostatic neoplasia OR prostatic tumor OR prostatic tumour OR prostate gland cancer OR prostate malignancy OR prostate malignant neoplasm OR prostatic cancer OR prostatic malignancy) | 270369 |
| #5 | "TS=(Radiomics OR Radiomic OR machine learning OR artificial intelligence OR Transfer Learning OR prediction model OR Deep learning OR ResNet OR AlexNet OR VGGNet OR GoogLeNet OR Ensemble Learning OR risk model OR risk score OR random forest OR neural network OR neural networks OR CNN OR K-Nearest Neighbor OR Support vector machine OR SVM OR Gradient Boosting Machine OR Nomogram OR XGBoost OR Adaboost OR LightGBM OR CatBoost OR Gradient Boosting OR Decision tree OR Regression Trees OR Naive Bayesian OR Multilayer perceptron OR Bayesian network) | 3555196 |
| #6 | "TS=(Lymphatic Metastasis OR Lymphatic Metastases OR Lymph Node Metastasis OR Lymph Node Metastases OR Lymph node invasion OR Lymph node involvement OR lymph gland metastases OR lymph gland metastasis OR ymph metastases OR lymph metastasis OR lymph node positivity OR lymphoid metastasis) | \| 115395 \| \| --- \| |
| #7 | "#4 AND #5 AND #6 | 1528 |

**
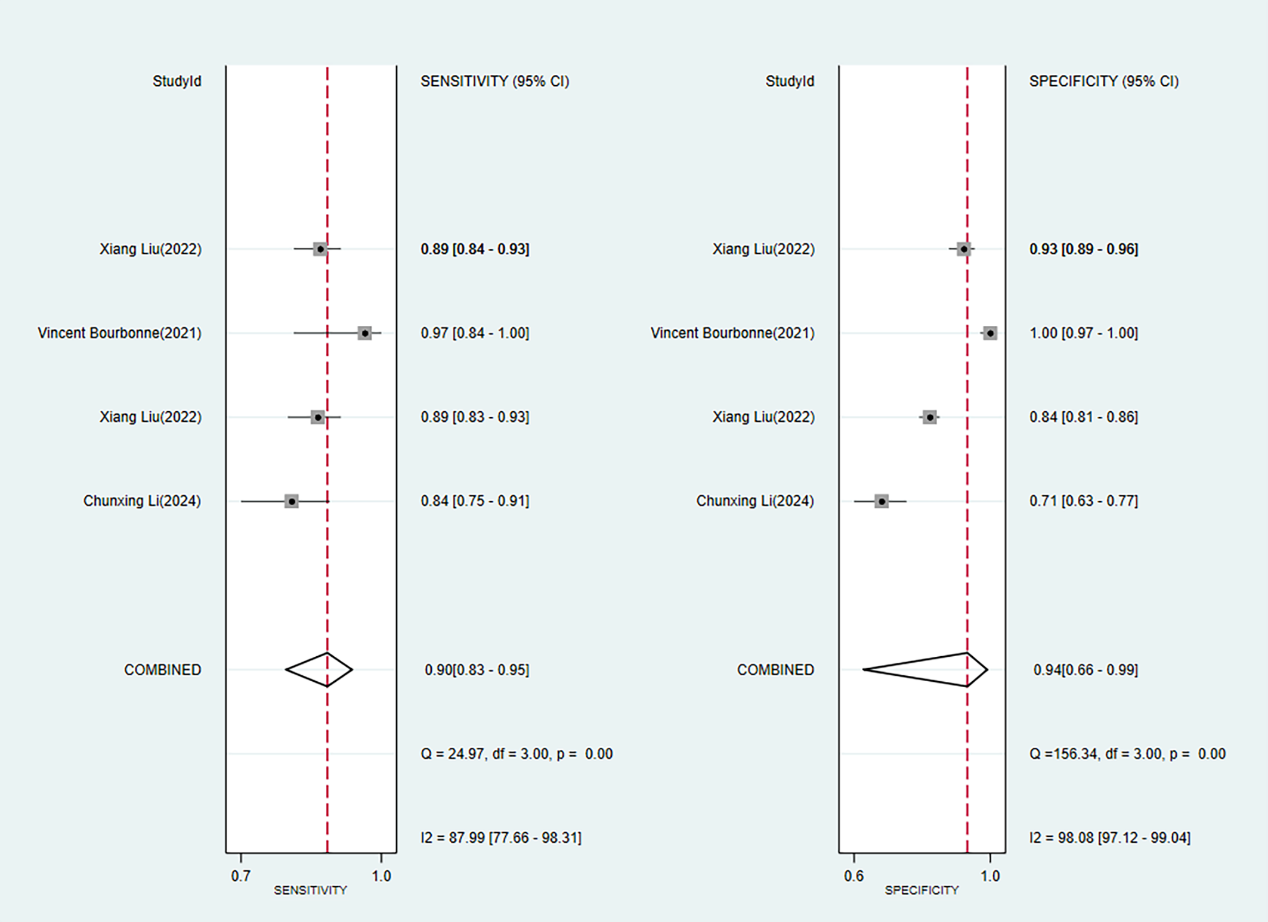
**

**Figure S1.** Forest plot of SEN and SPC for MRI radiomics-based ML in detecting LNM.

**
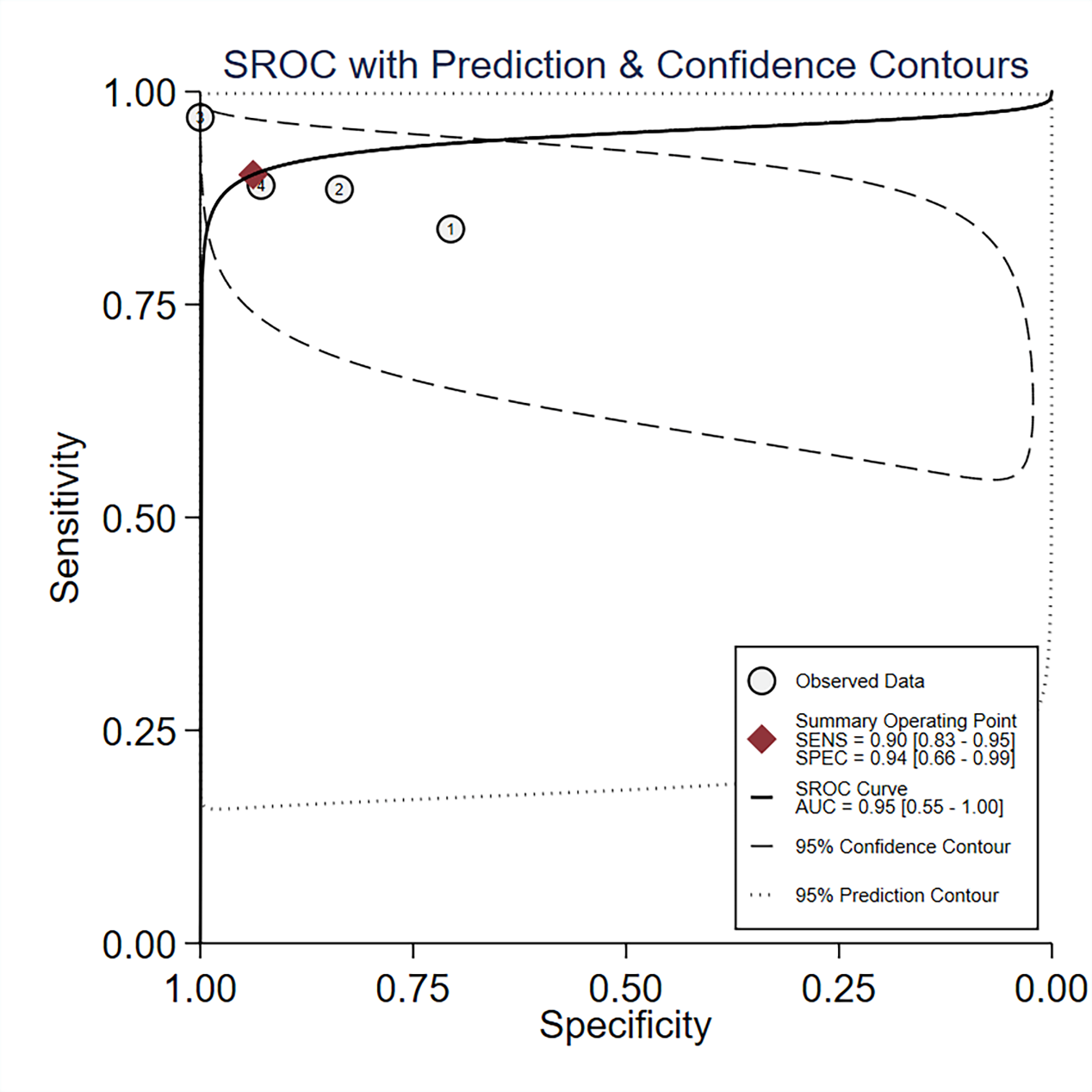
**

**Figure S2.** SROC of MRI radiomics-based ML.

**
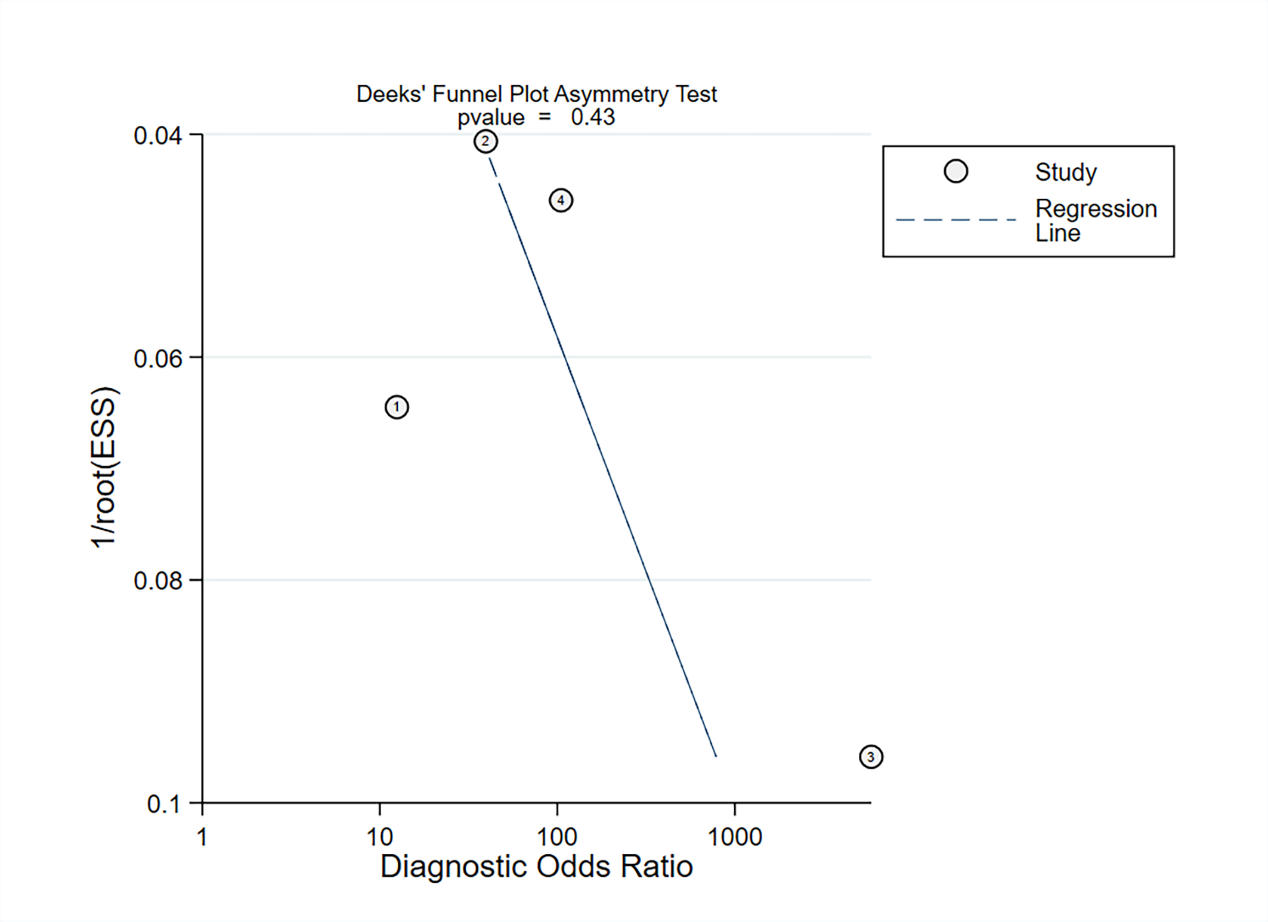
**

**Figure S3.** Deeks’ funnel plot of MRI radiomics-based ML.

**
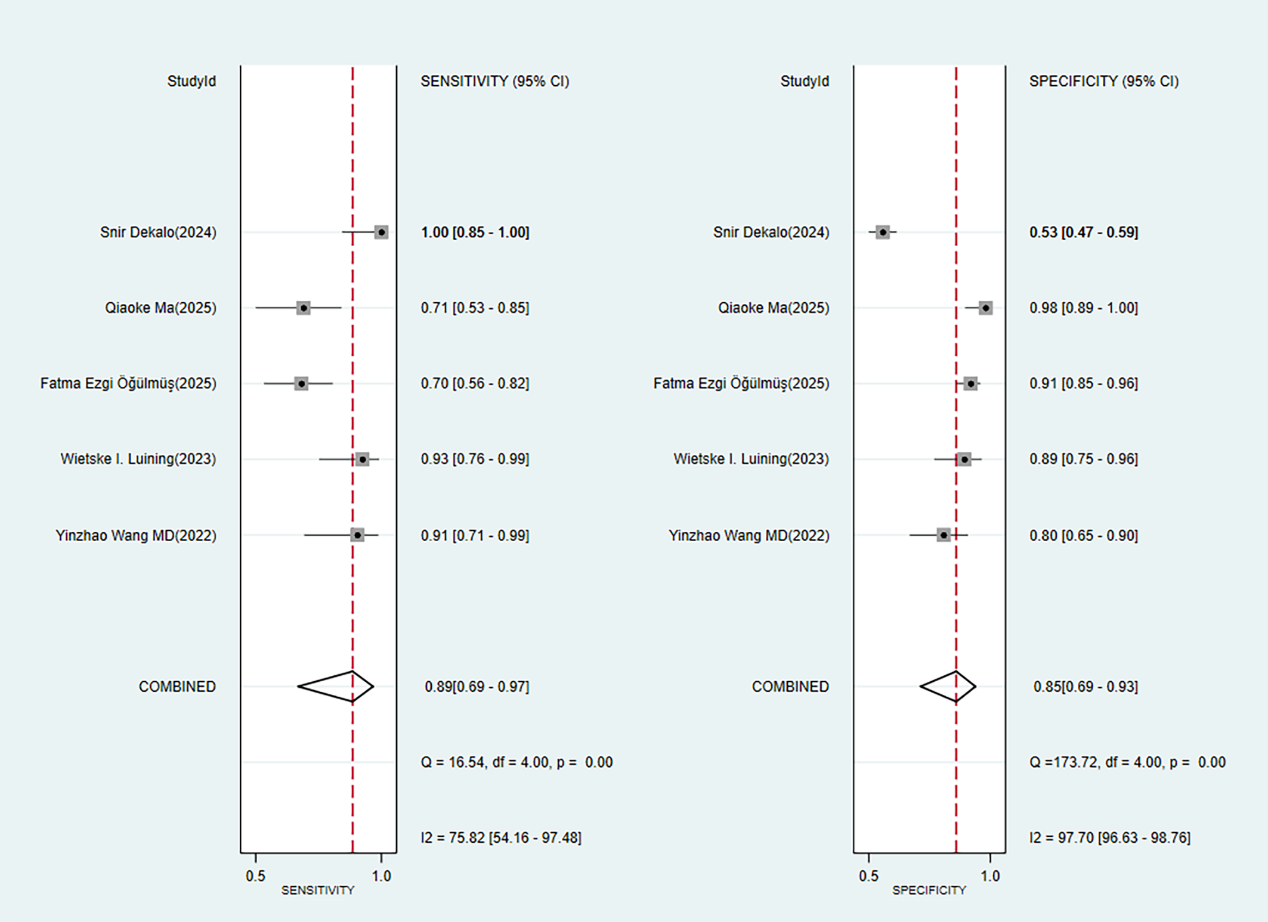
**

**Figure S4.** Forest plot of SEN and SPC for PET/CT radiomics-based ML in detecting LNM in the training cohort.

**
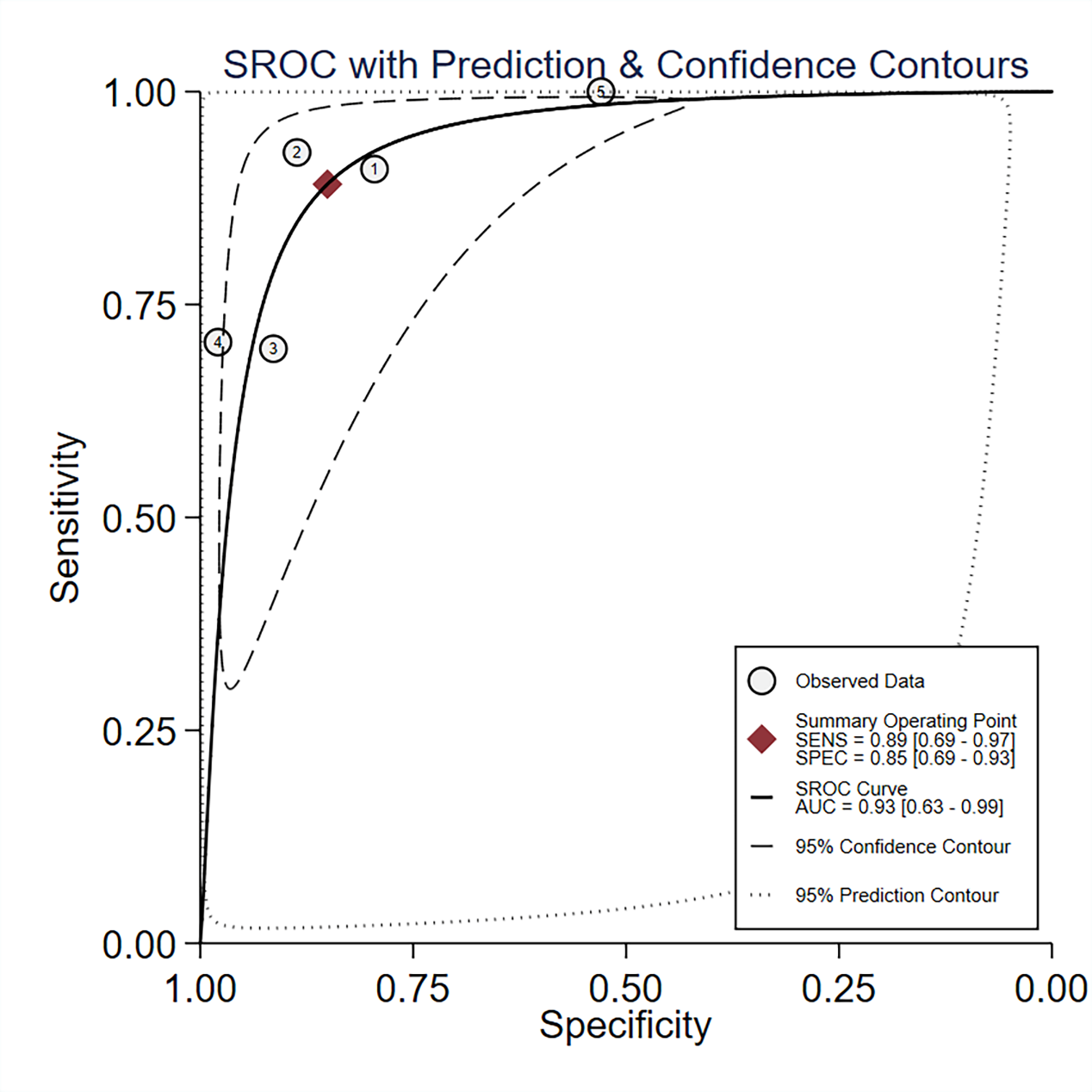
**

**Figure S5.** SROC of PET/CT radiomics-based ML.

**
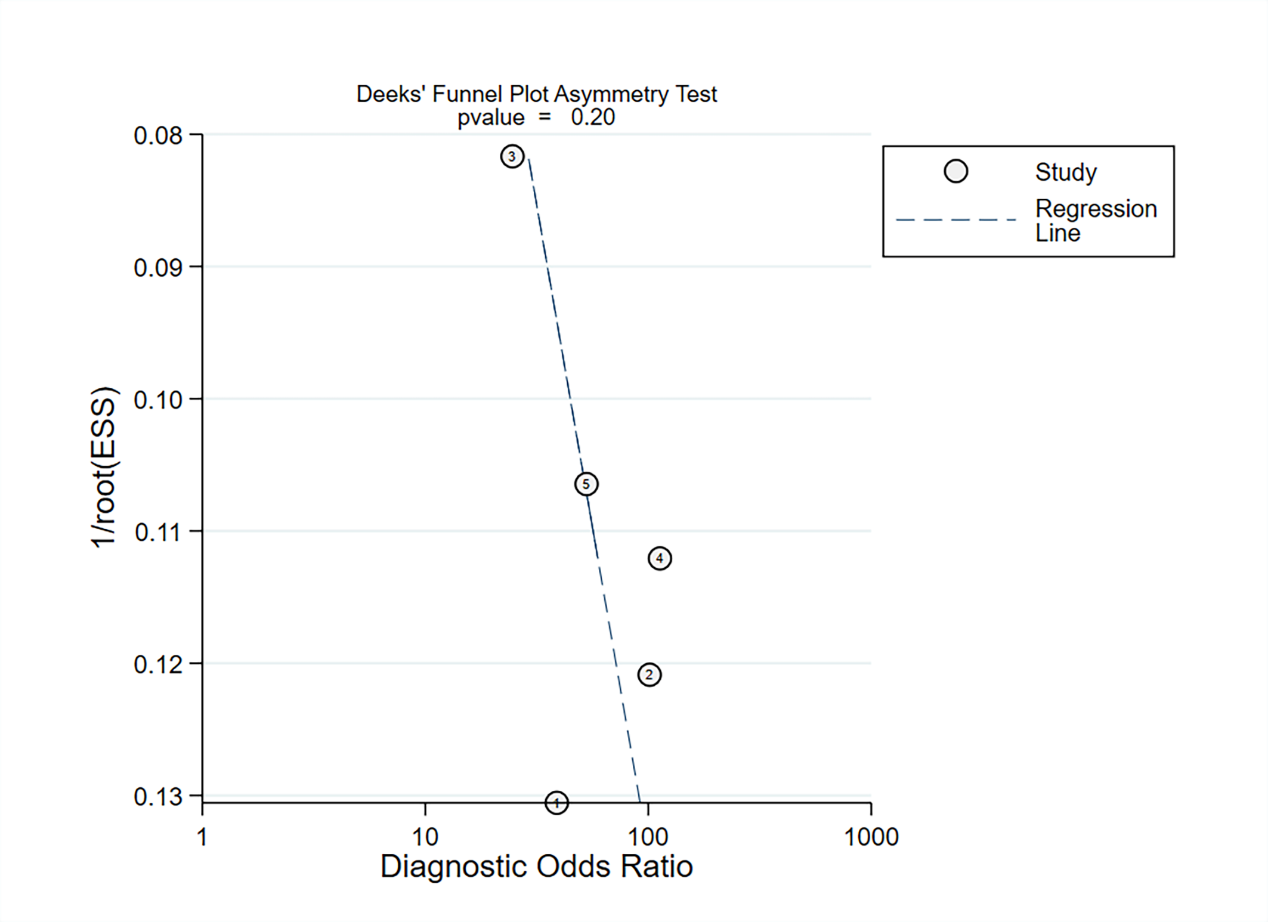
**

**Figure S6.** Deeks’ funnel plot of PET/CT radiomics-based ML.

**
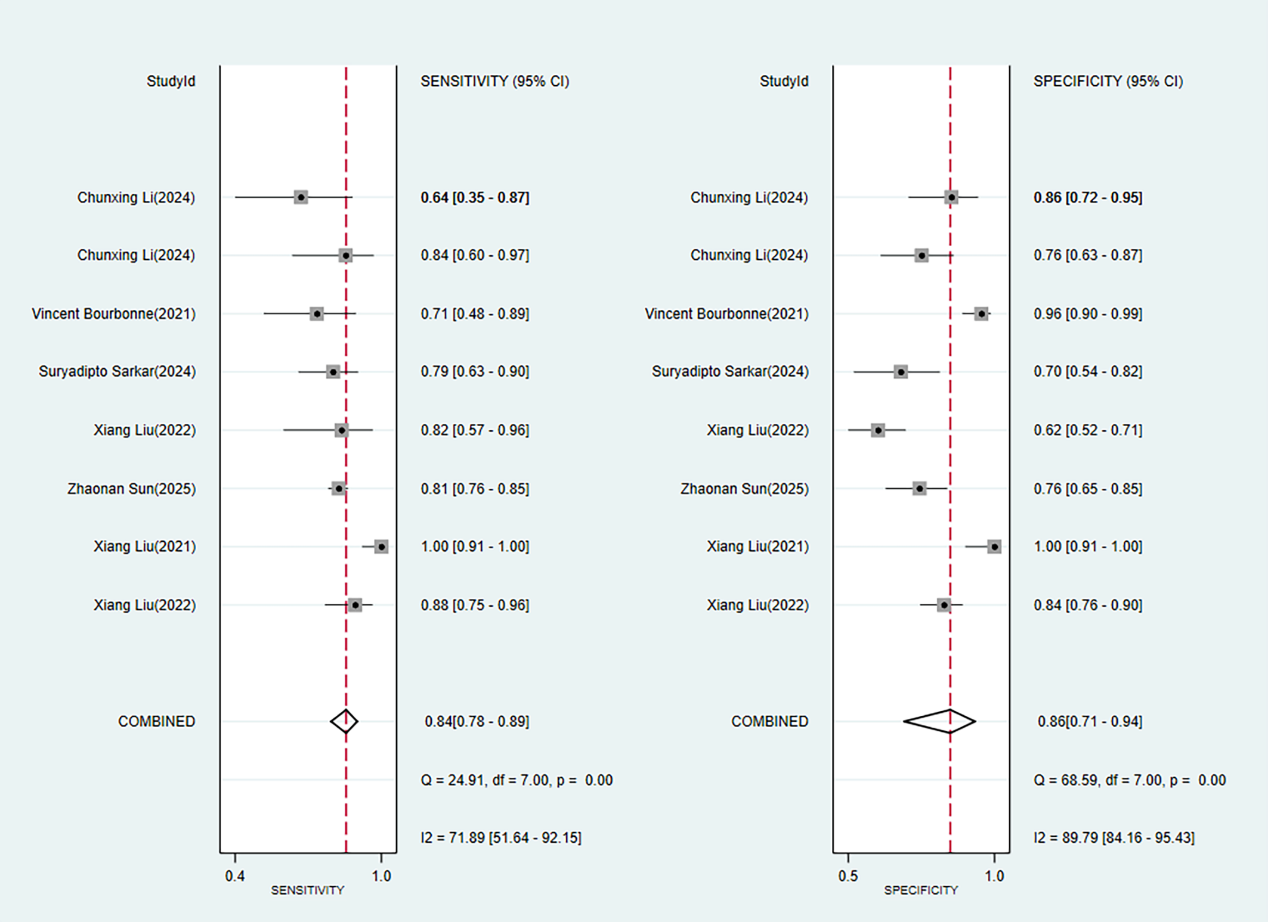
**

**Figure S7.** Forest plot of SEN and SPC for MRL radiomics-based ML in detecting LNM in the validation set.

**
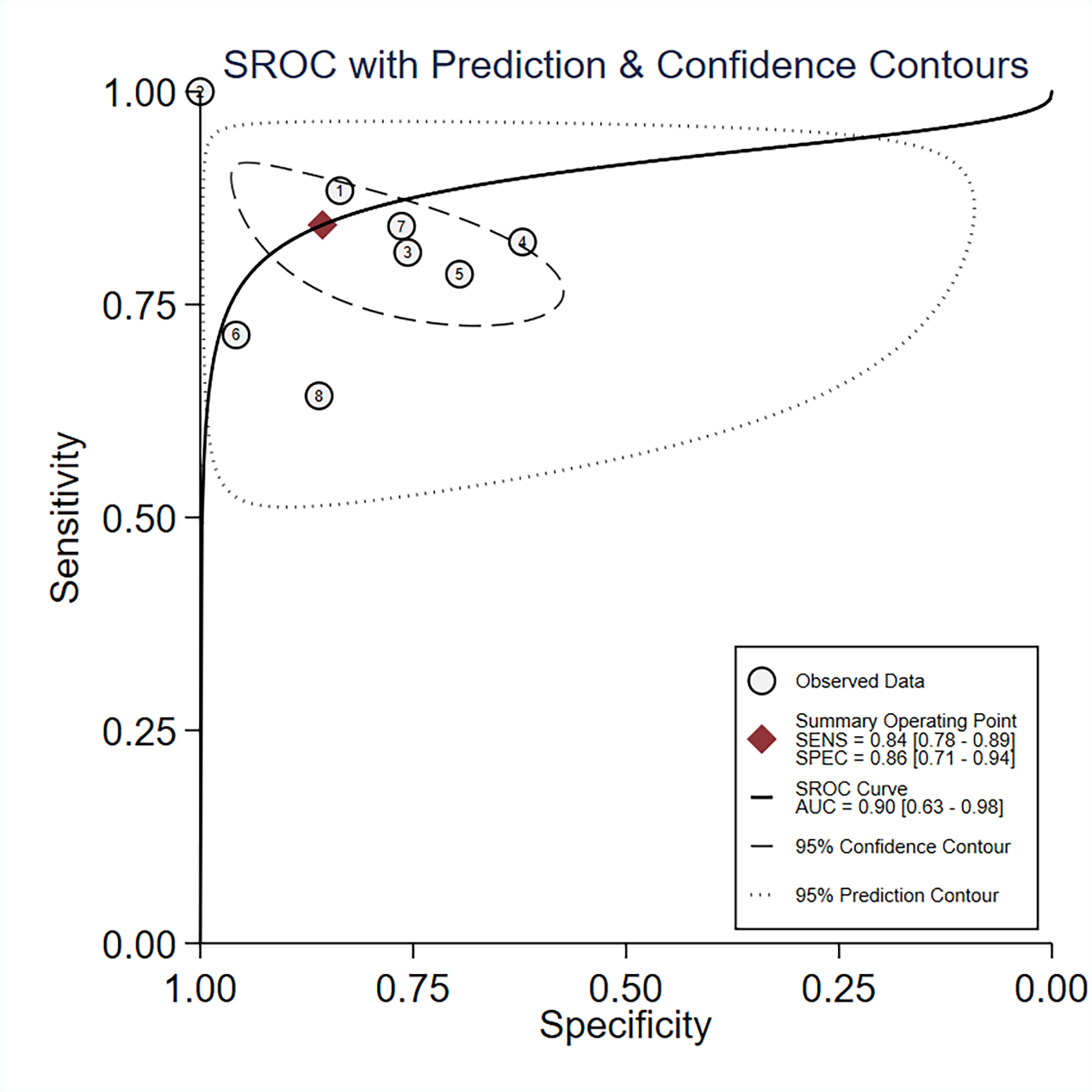
**

**Figure S8.** SROC curve of MRL radiomics-based ML.

**
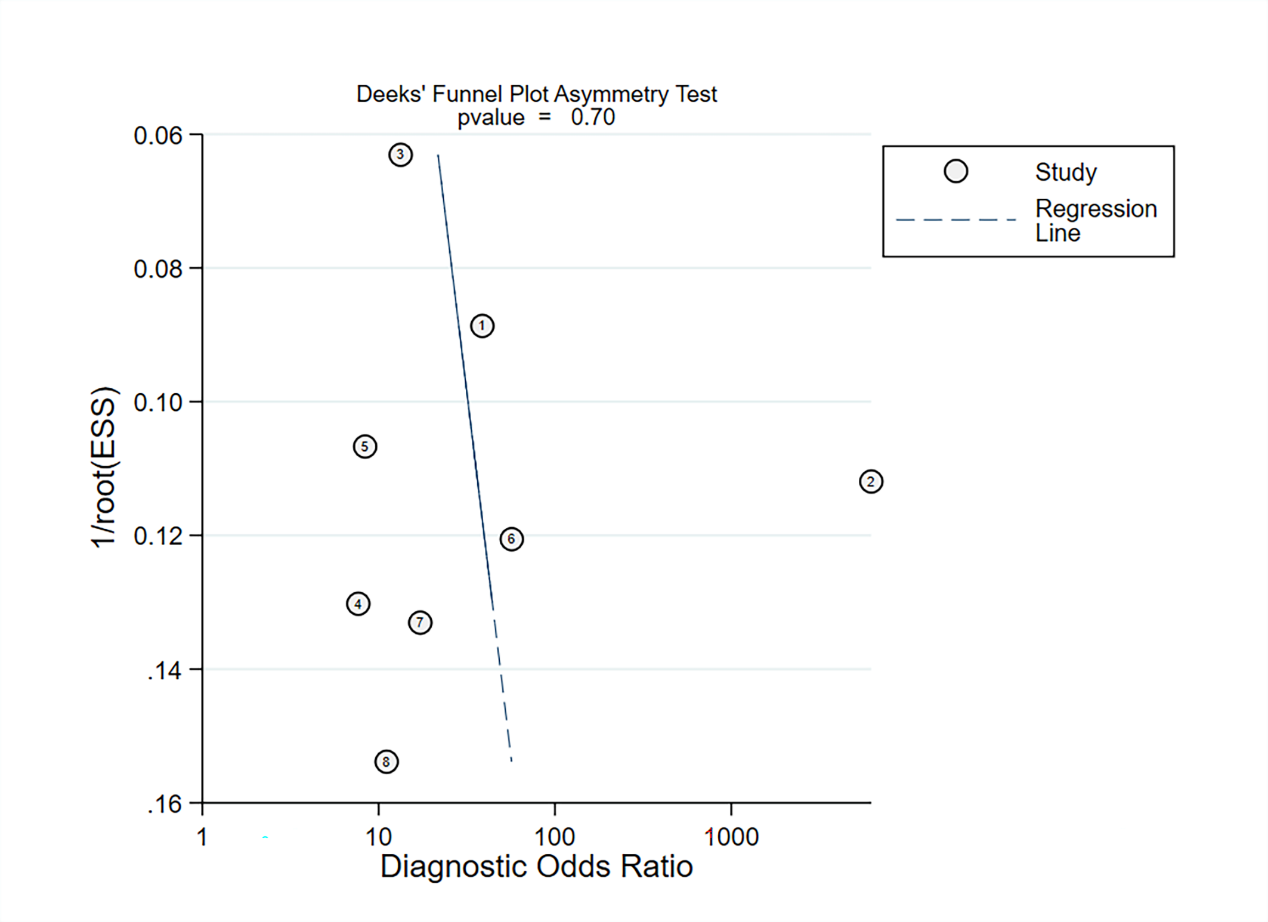
**

**Figure S9.** Deeks’ funnel plot of MRL radiomics-based ML.

**
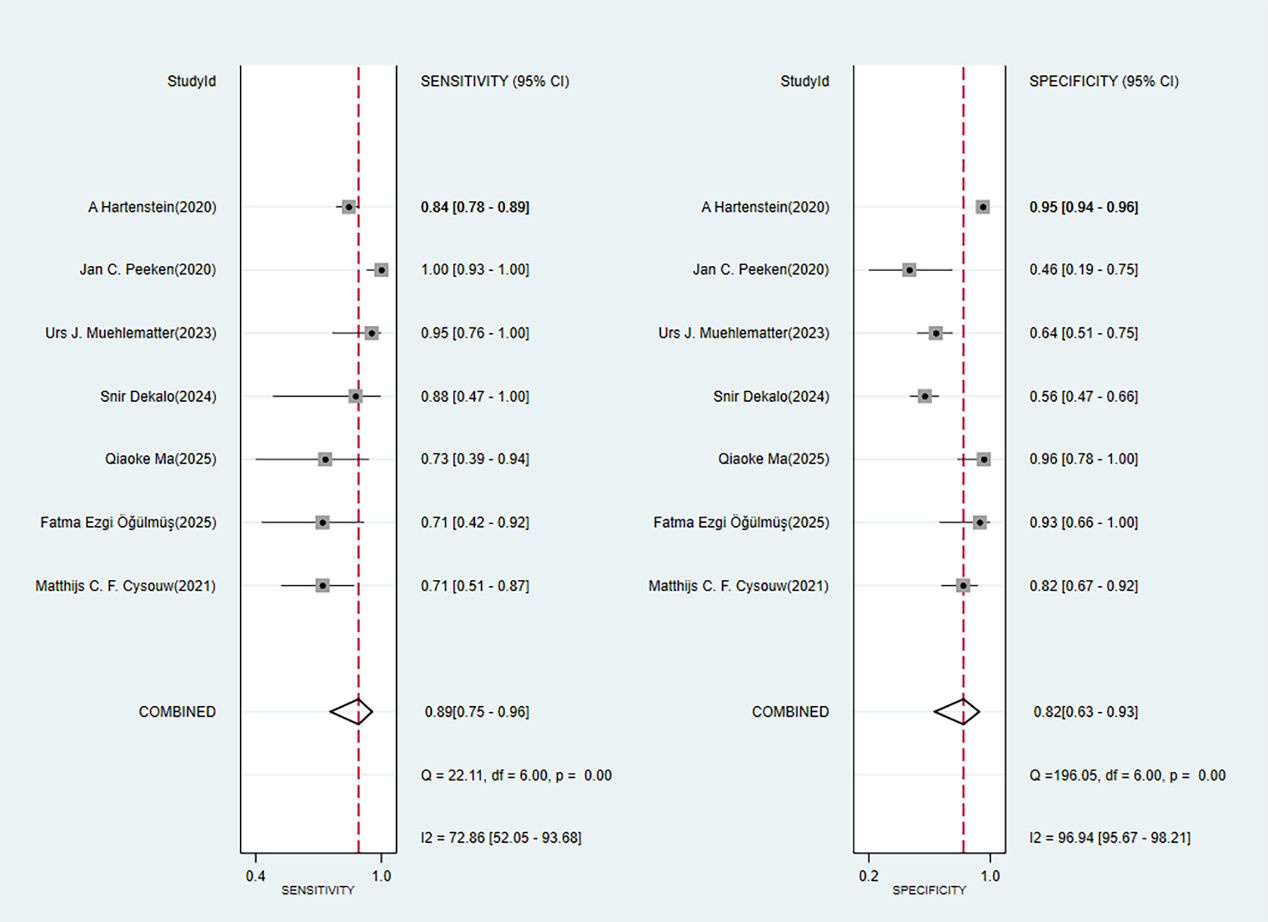
**

**Figure S10.** Forest plot of SEN and SPC for PET/CT radiomics-based ML in the validation set (PET/CT subgroup).

**
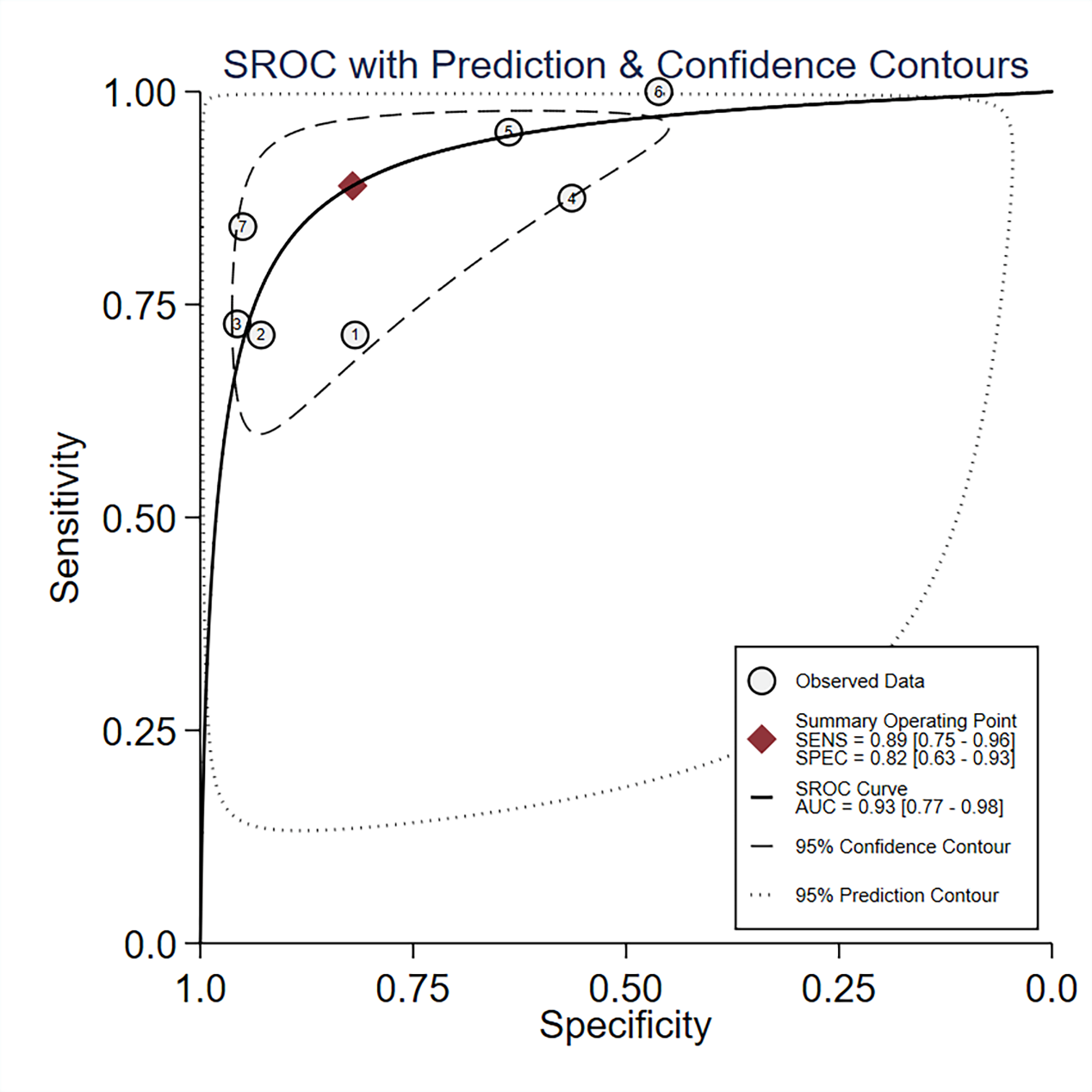
**

**Figure S11.** SROC curve of PET/CT radiomics-based ML (PET/CT subgroup).

**
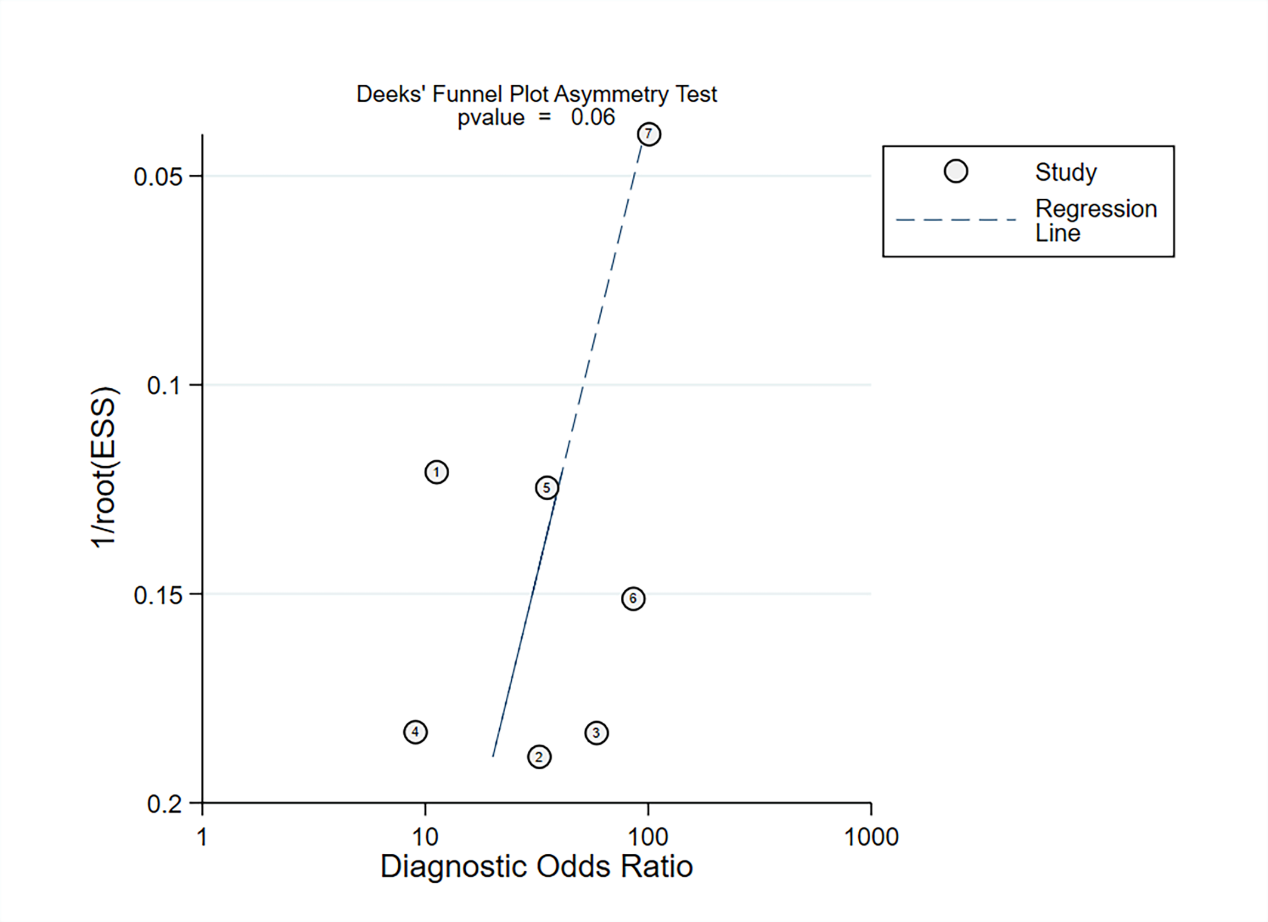
**

**Figure S12.** Deeks’ funnel plot of PET/CT radiomics-based ML (PET/CT subgroup).

**
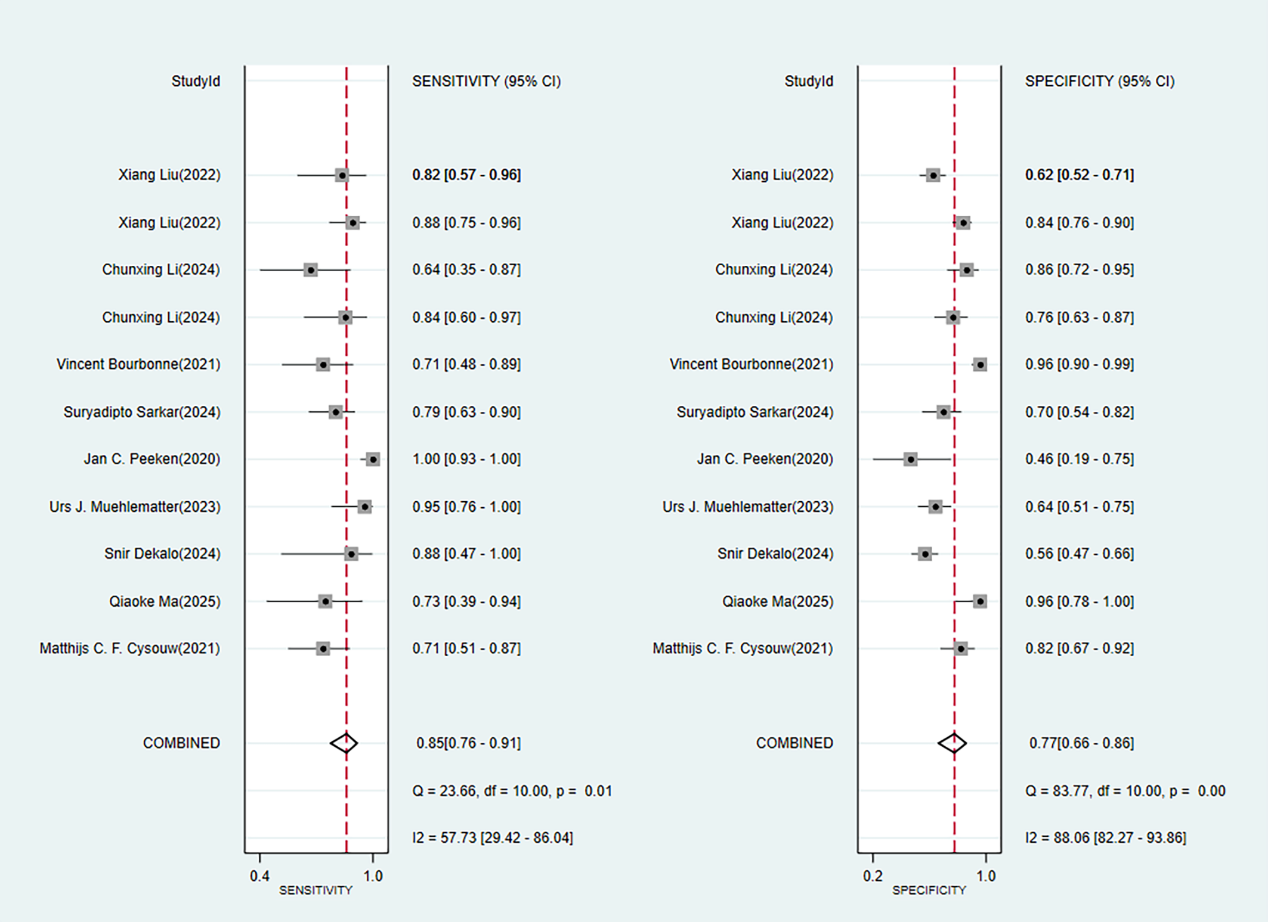
**

**Figure S13.** Forest plot of SEN and SPC for radiomics-based ML (ML subgroup).

**
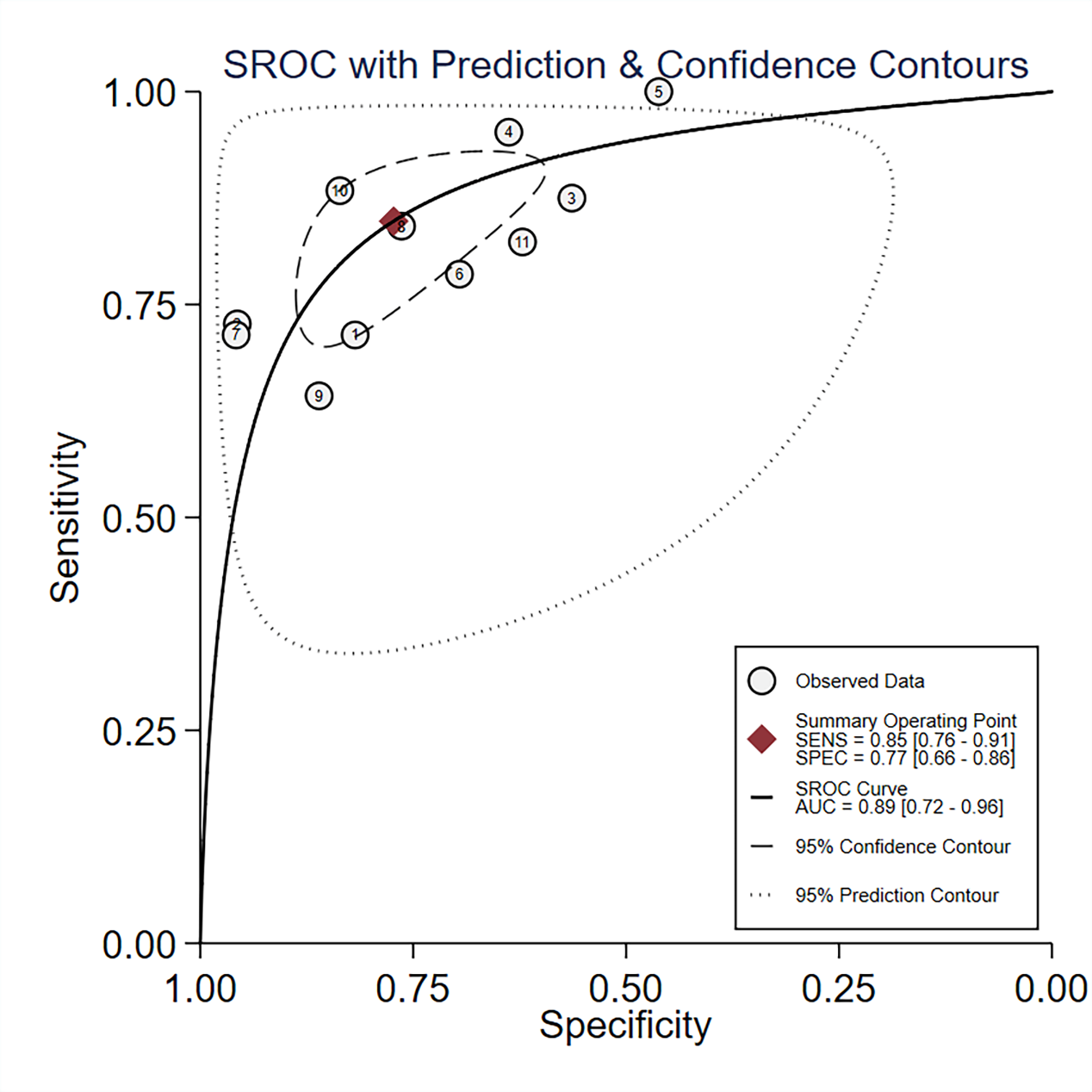
**

**Figure S14.** SROC curve of radiomics-based ML (ML subgroup).

**
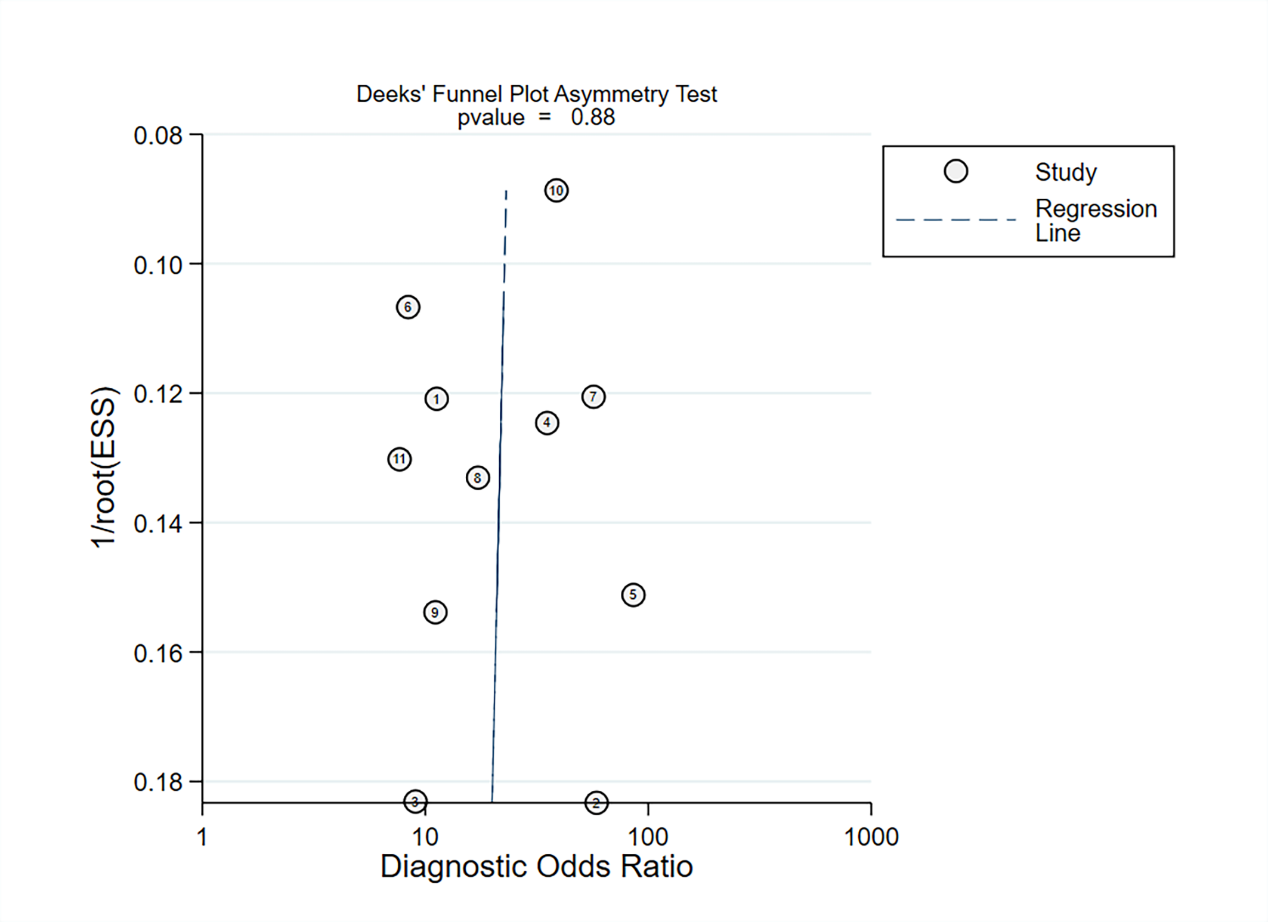
**

**Figure S15.** Deeks’ funnel plot of radiomics-based ML(ML subgroup).

**
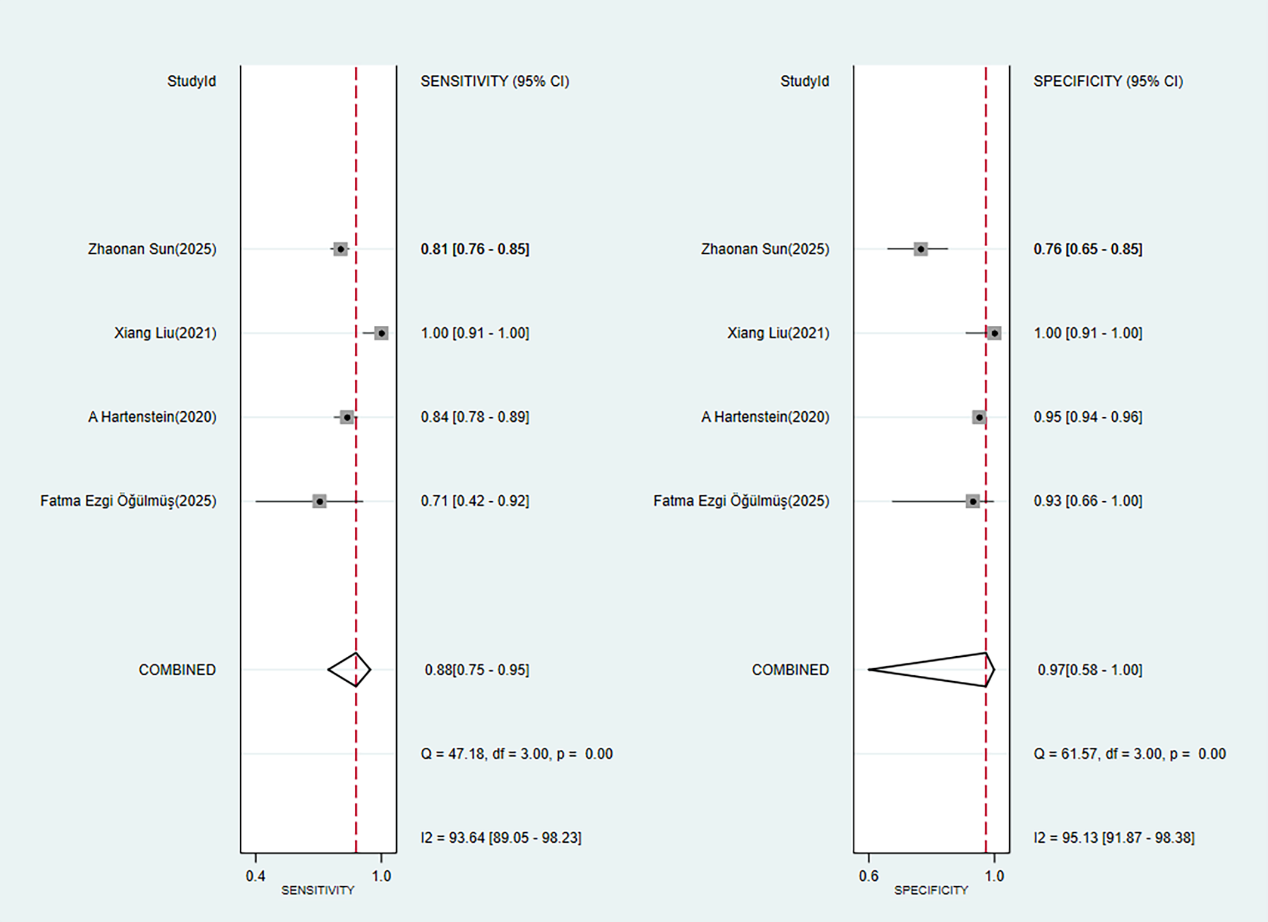
**

**Figure S16.** Forest plot of SEN and SPC for radiomics-based DL in detecting LNM in the validation set.

**
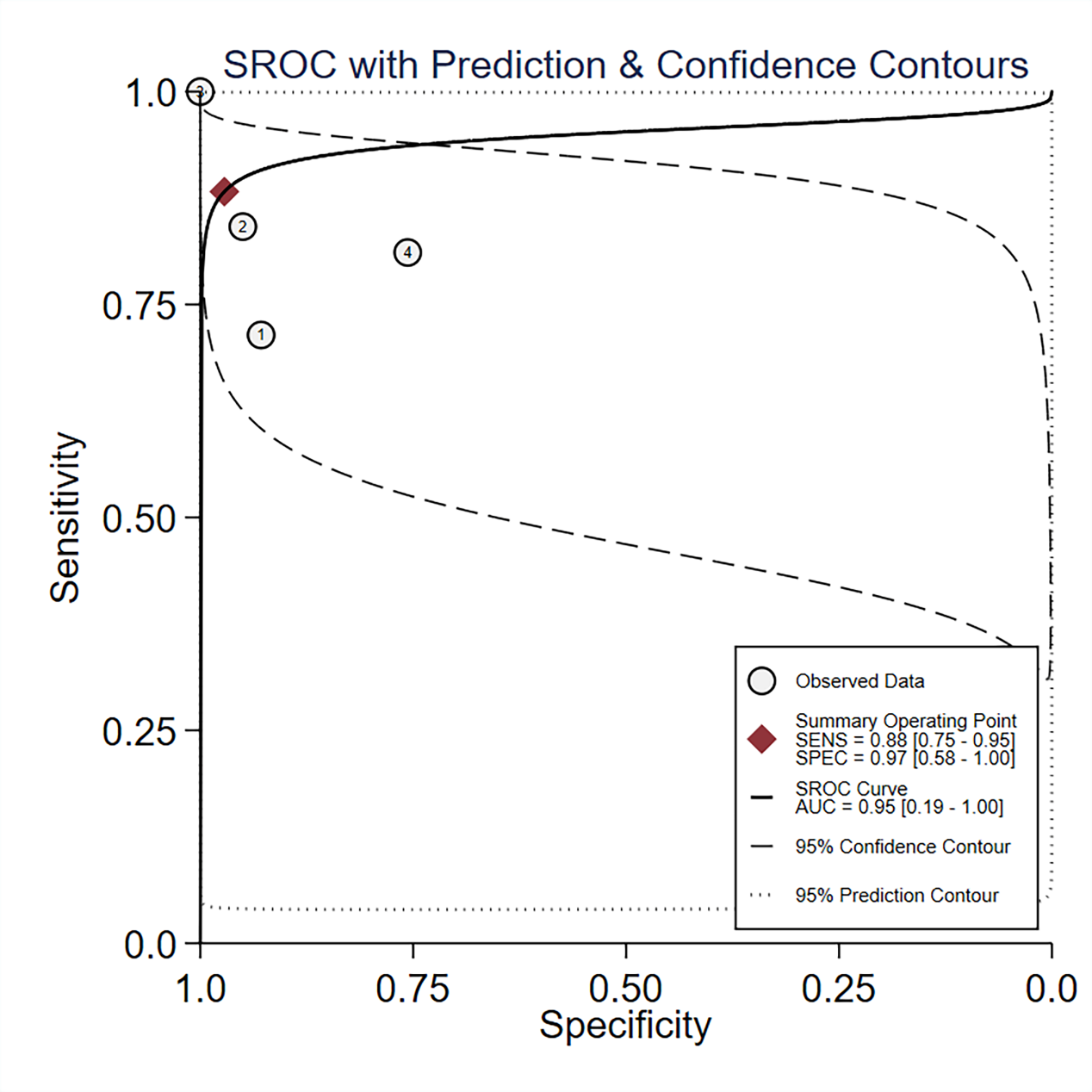
**

**Figure S17.** SROC curve of radiomics-based DL.

**
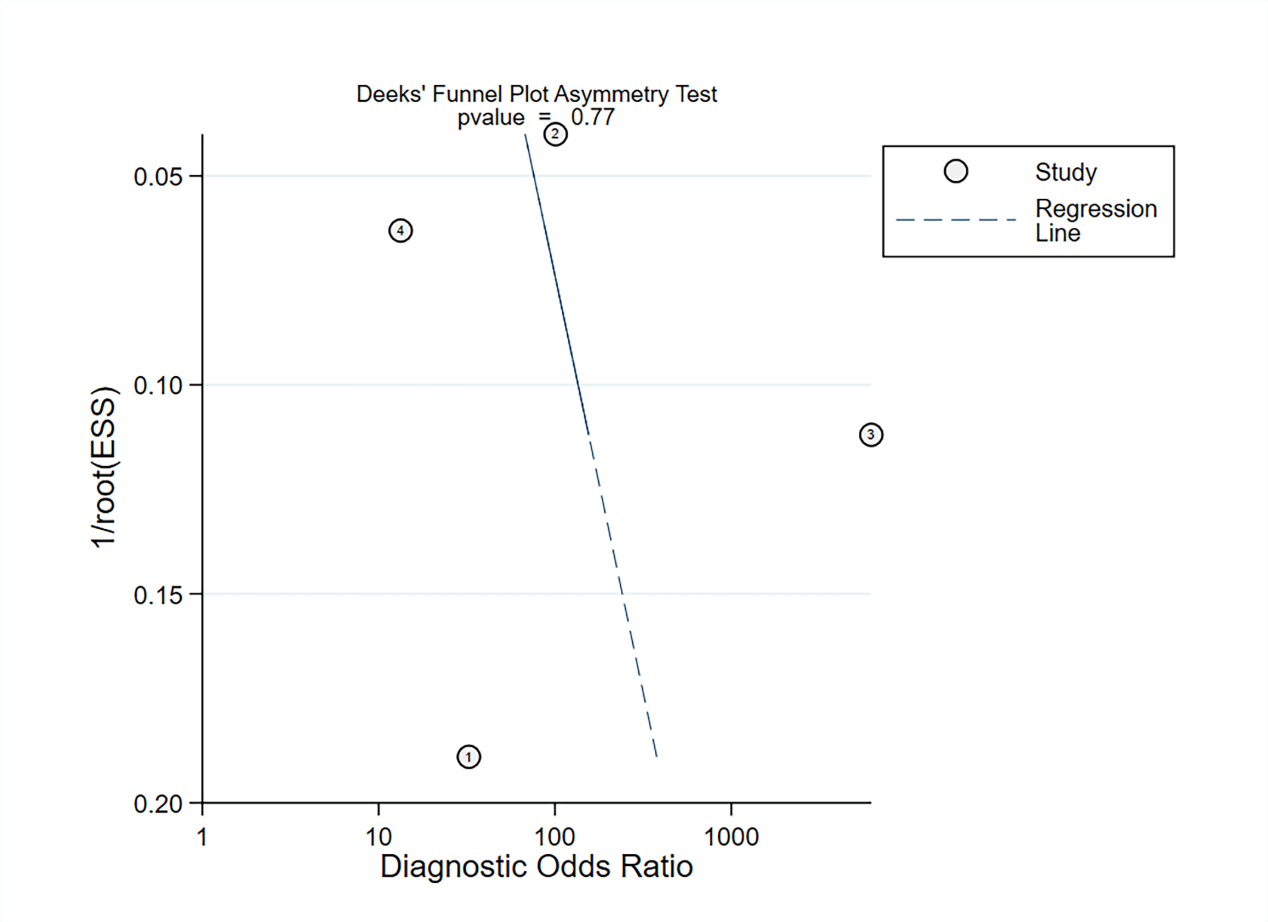
**

**Figure S18.** Deeks’ funnel plot of radiomics-based DL.
